# Long-Lasting Event-Related Beta Synchronizations of Electroencephalographic Activity in Response to Support-Surface Perturbations During Upright Stance: A Pilot Study Associating Beta Rebound and Active Monitoring in the Intermittent Postural Control

**DOI:** 10.3389/fnsys.2021.660434

**Published:** 2021-05-21

**Authors:** Akihiro Nakamura, Yasuyuki Suzuki, Matija Milosevic, Taishin Nomura

**Affiliations:** Department of Mechanical Science and Bioengineering, Graduate School of Engineering Science, Osaka University, Osaka, Japan

**Keywords:** upright posture, postural control, electroencephalogram, event-related synchronization, beta rebound, intermittent control

## Abstract

Movement related beta band cortical oscillations, including beta rebound after execution and/or suppression of movement, have drawn attention in upper extremity motor control literature. However, fewer studies focused on beta band oscillations during postural control in upright stance. In this preliminary study, we examined beta rebound and other components of electroencephalogram (EEG) activity during perturbed upright stance to investigate supraspinal contributions to postural stabilization. Particularly, we aimed to clarify the timing and duration of beta rebound within a non-sustained, but long-lasting postural recovery process that occurs more slowly compared to upper extremities. To this end, EEG signals were acquired from nine healthy young adults in response to a brief support-surface perturbation, together with the center of pressure, the center of mass and electromyogram (EMG) activities of ankle muscles. Event-related potentials (ERPs) and event-related spectral perturbations were computed from EEG data using the perturbation-onset as a triggering event. After short-latency (<0.3 s) ERPs, our results showed a decrease in high-beta band oscillations (event-related desynchronization), which was followed by a significant increase (event-related synchronization) in the same band, as well as a decrease in theta band oscillations. Unlike during upper extremity motor tasks, the beta rebound in this case was initiated before the postural recovery was completed, and sustained for as long as 3 s with small EMG responses for the first half period, followed by no excessive EMG activities for the second half period. We speculate that those novel characteristics of beta rebound might be caused by slow postural dynamics along a stable manifold of the unstable saddle-type upright equilibrium of the postural control system without active feedback control, but with active monitoring of the postural state, in the framework of the intermittent control.

## Introduction

Supraspinal contributions to postural stabilization during human upright stance have been demonstrated by postural instability in patients with neurological disorders such as Parkinson’s disease (PD), multiple sclerosis, and stroke (e.g., Horak et al., 1992; Frzovic et al., 2000; Geurts et al., 2005; Visser et al., 2008; Perera et al., 2018; Suzuki et al., 2020). Because impairment of postural stability due to neurological diseases is one of the major quality-of-life factors (Sterling et al., 2001) and it has also been linked to individual fall risks (Zhou et al., 2017), understanding the supraspinal information processing for stabilizing upright stance is of crucial importance for our aging societies.

A traditional approach for characterizing the supraspinal control of upright posture is to examine alterations in patterns of postural sway and postural responses to perturbations induced by a cognitive load (Woollacott and Shumway-Cook, 2002) and by motor learning during adapting to novel environmental demands (Horak and Nashner, 1986), which provides a glimpse into the roles played by the supraspinal networks in the control of upright posture. Electroencephalogram (EEG) activity and/or motoneuronal responses to transcranial magnetic stimulation (TMS) during standing are more direct methods to characterize electrical activity of the cerebral cortex associated with postural control. For example, using EEG recordings, it has been shown that postural reactions during voluntary postural sway are triggered by the central command mechanisms associated with bursts of gamma-band activities (Slobounov et al., 2005). Moreover, soleus muscle showed larger motor evoked potentials in response to TMS stimuli during standing on a continuously or impulsively moving planform, compared to standing on a still platform, which implies increased corticospinal excitability in more challenging postural environments (Solopova et al., 2003; Taube et al., 2006). Yet, there is relatively limited knowledge on how activities of the cerebral cortex, particularly those measured by EEG, encode sensory information processing and motor control during stabilization of upright stance (Jacobs and Horak, 2007; Bolton, 2015; Wittenberg et al., 2017).

Electroencephalogram signals during upright stance have traditionally been investigated using event-related potentials (ERPs) in response to brief postural perturbations, where a perturbation-event-locked average of EEG time-series is computed to achieve high signal-to-noise ratio. Particularly, the most representative ERP identified in EEG activities in postural response to brief mechanical perturbations has a negative potential and it is referred to as the N1 (Quant et al., 2005; Varghese et al., 2017). The N1 potential is typically induced with a latency of about 90–170 ms and amplitudes ranging from −10 to −70 μV, while the responses are spatially distributed over the frontal, central, and parietal cortices (Varghese et al., 2017). There are several interpretations for the origins of the N1 responses. One considers that N1 represents neural processing of sensory information (Dietz et al., 1984; Dimitrov et al., 1996; Staines et al., 2001) necessary for coordinating reactive balance responses, based on the fact that the latency and amplitude of N1 are altered by the afferent transmission delay (Dietz et al., 1985) and the level of cognitive load (Quant et al., 2004). Another interpretation is that N1 represents an error signal for detecting postural instability (Adkin et al., 2006, 2008; Mochizuki et al., 2009; Payne et al., 2019). Payne et al. have been working to characterize the N1 and other ERPs during perturbed stance in recent years. They showed that: (1) people with lower balance ability exhibited larger N1 responses compared to those with better balance control (Payne and Ting, 2020); and (2) N1 includes startle responses in addition to balance-correcting motor responses (Payne et al., 2018). Taken together, these studies provide insights into specific cortical responses to balance perturbations during upright stance.

Time-frequency characteristics of EEG responses induced by mechanical perturbations to the upright stance can also be analyzed by computing event-related spectral perturbations (ERSPs) that represent event-triggered EEG both for phase-locked and phase-unlocked components (Slobounov et al., 2005; Smith et al., 2012; Varghese et al., 2014, 2015; Mierau et al., 2017; Ozdemir et al., 2018; Peterson and Ferris, 2018; Solis-Escalante et al., 2019). Note that the EEG components, which appear in ERPs as the phase-locked components, also appear in ERSPs, while the inverse is not always true. ERSP represents neural oscillations at distinct frequency-bands to characterize the balance between neuronal excitation and inhibition (Buzsáki et al., 2012; Muthukumaraswamy, 2014). Using EEG ERSP, it may be possible to identify cortical responses to a particular event during standing, as shown during other motor and cognitive tasks (Makeig, 1993; Pfurtscheller and Lopes da Silva, 1999). For motor tasks other than the upright posture, it is considered that low-frequency band-limited cortical synchronizations (<20 Hz) are associated with a deactivated state of the corresponding networks, and that high-frequency band-limited cortical synchronizations (>20 Hz) reflect a state of active information processing in the sensorimotor area (Pfurtscheller and Lopes da Silva, 1999). Particularly, in the sensorimotor cortex, event-related desynchronization (ERD) at beta band (13–30 Hz) prior to and/or during motor execution, as well as event-related synchronization (ERS) of beta band oscillations after the movement are well known (Pfurtscheller et al., 1996; Pfurtscheller and Lopes da Silva, 1999). Beta band ERS is typically referred to as the post-movement rebound or simply as beta rebound, and it might be associated with *status quo* in terms of postural maintenance of upper extremities (Engel and Fries, 2010) and afferent sensory information processing (Cassim et al., 2001). Attenuation of the beta rebound in PD patients is consistent with impaired sensory integration (Vinding et al., 2019). Moreover, beta rebound is typically observed in Go/NoGo tasks for upper extremities, with and without motor executions, which is also a typical movement-related EEG response characteristic (Alegre et al., 2004; Zhang et al., 2008; Swann et al., 2009). Since beta ERS for the NoGo response is not accompanied with an actual motor execution, it may represent motor-related decision-making processing in the supraspinal networks, in addition to information processing of sensory feedback signals. Therefore, considering attenuated beta ERS responses reported for the Go/NoGo task in PD patients (Wu et al., 2019), the beta ERD and the subsequent beta ERS (beta rebound) represent information processing performed by the cortico-basal ganglia motor loop.

Among a small number of studies analyzing ERSPs during upright stance, Varghese et al. (2014) reported that whole-body perturbations during upright stance caused phase-locked ERS in theta (4–7 Hz), alpha (8–12 Hz), and beta bands, within very short response latencies (at most a few 10 ms), which correspond to the time range of the N1 potentials. Smith et al. (2012) demonstrated that PD patients exhibit greater beta ERD after a cue, but prior to a predictable small-magnitude perturbation, compared to healthy control participants in the Cz electrode. Similarly, in the lower extremities study during sitting, Walker et al. (2020) reported that beta ERD and ERS appear after the stimulation to rotate the ankle, where the beta ERD was enhanced in the elderly, compared to young people. In addition, some studies showed that brief mechanical perturbations applied by pulling the body during standing posture induced beta ERD after the theta and alpha ERSs with short latencies (Peterson and Ferris, 2018). However, beta rebound, which has been examined extensively during voluntary and simple reactive motor tasks in upper extremities, has not been investigated during postural control tasks in upright stance. One of the key issues in dealing with postural recovery processes during upright standing in response to external perturbations is the difference in the time-scale of mechanical dynamics compared to movements of the upper extremities. Namely, postural responses of musculoskeletal system during upright stance typically persist over longer periods of time (i.e., a few seconds), unlike those of the upper extremities that are typically completed within a few 100 ms.

In this study, we therefore examined ERSPs during the long-lasting postural recovery process of upright equilibrium in response to small impulsive (step-like) support-surface perturbations. Particular interest was to investigate the timing and the duration of beta band ERD and ERS responses, and to quantify them. Because a postural recovery process from a perturbed posture to the upright equilibrium is transient, and it settles down eventually to the post-recovery equilibrium state, the post-recovery state could be regarded as a *status quo* for postural maintenance. Therefore, similarly to the upper limbs (Engel and Fries, 2010), we hypothesized that beta ERD during the recovery response and the subsequent post-movement (post-recovery) beta rebound (beta ERS) would be present in the EEG cortical activities following upright stance perturbations. If we could observe beta ERD and beta rebound as hypothesized, the next objective would be to clarify in the temporal profiles of the appearance of beta activities within the long-lasting biomechanical postural recovery response. In the simplest possible situation, beta ERD would appear persistently during the postural response while the muscles are active, and then a beta rebound would appear after the postural response is completed, i.e., as a post-movement rebound after a few seconds required for the upright posture to be fully recovered. This scenario is based on beta ERD and ERS during upper extremity tasks (Engel and Fries, 2010), with possible variations in time intervals due to the longer-lasting postural response. However, if we observe beta ERD and ERS with qualitative differences in temporal characterizations from those during upper extremity tasks, they could lead to shedding new light on mechanistic causes of the beta rebound. Indeed, the final phase of the postural recovery has a particular meaning for the intermittent control hypothesis that has received attention in recent years (Bottaro et al., 2008; Asai et al., 2009; Xiang et al., 2018; Suzuki et al., 2020), as elaborated in the next section. That is, the final phase of postural recovery takes place with a small postural tilt, during which, according to the intermittent control hypothesis, the active feedback control is switched off, and the postural state point approaches slowly the upright posture along a stable manifold of the unstable saddle-type upright equilibrium in the state space of postural control system. Because the switching action of the active feedback control, such as deciding to maintain the switch off or to turn the switch on, would involve active information processing, we expected to find cortical activities associated with it.

## Theoretical Background

In this section, we overview fundamental neuromechanics of upright posture during quiet stance and those in response to a support-surface perturbation, based on the intermittent control model (Bottaro et al., 2008; Asai et al., 2009; Nomura et al., 2020; Suzuki et al., 2020) to clarify the perspectives of this study. To this end, we consider an inverted pendulum model of human upright stance, stabilized by an intermittent feedback controller. In the latter half of this section, we associate neural mechanisms of the intermittent ON-OFF switching of an active feedback controller for stabilizing upright stance with those of action selection in the Go-NoGo tasks, by which we illustrate a model-based motive for exploring the EEG beta rebound in a time span of a few seconds after perturbing quiet stance.

### Model

We consider a triple-rigid-link inverted pendulum, composed of the first link representing feet (Foot link), the second link representing left and right lower extremities (LE link), and the third link representing head-arm-trunk segments (HAT link), which are connected by hinge joints, corresponding to the ankle and the hip joints. See Morasso et al. (2019) and Supplementary Materials (Supplementary Figure 1 and Supplementary Sections 1–6, which is also available at https://doi.org/10.5281/zenodo.3955495). The Foot link is assumed to be fixed on the horizontal support-surface, i.e., we assume that toe and heel of the Foot link are always in contact with the support-surface with no slips. Thus, postural sway during quiet stance and perturbed posture can indeed be described only by two degrees of freedom, i.e., the ankle joint angle θ_a_ and the hip joint angle θ_h_, for the double inverted pendulum (DIP) with the LE and the HAT links by the following equation of motion (Suzuki et al., 2012; Morasso et al., 2019).

(1) $$M\left(\theta \right)\dot{\theta }+C\left(\theta ,\dot{\theta }\right)+G\left(\theta \right)=T^{\text{pass}}+T^{\text{act}}+T^{\text{reflex}}+T^{\text{pert}}+T^{\text{n}}$$

where θ = (θ_a_, θ_h_)^*T*^, and *M*(θ), $C\left(\theta ,\dot{\theta }\right)$ and *G*(θ) are the inertia matrix, the term of centrifugal and Coriolis forces and the gravitational toppling torque. Mathematical expressions of these terms are defined in Supplementary Section 6 of Supplementary Materials. $T^{\text{pass}}={\left(\tau_{\text{a}}^{\text{pass}},\tau_{\text{h}}^{\text{pass}}\right)}^{T}$ represents the passive joint torque, which is determined by the torsional elasticity and viscosity of the ankle joint (*K*_a_ and *B*_a_) and those of the hip joint (*K*_h_ and *B*_h_) for a given set of constant muscle tonuses that are determined in a feedforward manner for quiet stance. That is, *T*^pass^ is simply modeled by linear springs and dampers as follows.

(2) $$T^{\text{pass}}=\left(\begin{array}{c} \tau_{\text{a}}^{\text{pass}}\\ \tau_{\text{h}}^{\text{pass}} \end{array}\right)=-\left(\begin{array}{c} K_{\text{a}}\theta_{\text{a}}+B_{\text{a}}{\dot{\theta }}_{\text{a}}\\ K_{\text{h}}\theta_{\text{h}}+B_{\text{h}}{\dot{\theta }}_{\text{h}} \end{array}\right)$$

The intermittent control model assumes that the passive ankle stiffness (spring constant) *K*_a_ alone cannot stabilize the upright posture of the inverted pendulum (Loram and Lakie, 2002; Morasso and Sanguineti, 2002; Casadio et al., 2005). That is, *K*_a_ is smaller than the load stiffness representing the proportional constant for the linearized gravitational toppling torque *G*(θ). This means that time-delayed active feedback control is indispensable for stabilizing quiet stance. On the other hand, we assume large values of *K*_h_ and *B*_h_ in this study for simplicity, by which the hip joint angle is rigidly stabilized only by its passive viscoelasticity with no help of active feedback control (Morasso et al., 2019). $T^{\text{act}}={\left(\tau_{\text{a}}^{\text{act}},\tau_{\text{h}}^{\text{act}}\right)}^{T}$ represents active feedback control torque for stabilizing quiet stance. We assume that *T*^act^(*t*) at time *t* is determined by the supraspinal circuitry that processes time-delayed sensory feedback information on the posture θ(*t*−Δ) and $\dot{\theta }\left(t-\Delta \right)$, where Δ is a feedback time-delay for quiet stance (Δ = 200 ms). For simplicity, we assume that *T*^act^ is operated only at the ankle joint as $\tau_{\text{a}}^{\text{act}}$, i.e., $\tau_{\text{h}}^{\text{act}}=0$, regardless of the posture. See Suzuki et al. (2012) for a DIP model with active intermittent control on the hip as well as the ankle joints. *T*^pert^ and *T*^n^ represent the joint torques induced by the support-surface perturbation and endogenous motor noise, respectively. *T*^reflex^ represents a reflexive feedback control torque, which is operated in response to the perturbation only for a short duration at the early phase of postural recovery. For a given set of body parameters of the model, i.e., masses (*m*_Foot_, *m*_LE_, *m*_HAT_), link lengths, and local coordinate of the center of mass (CoM) for each of three links (see Supplementary Sections 1–3 of Supplementary Materials), we can identify the following physical quantities, such as positions of CoM, as a function of the joint angles at time *t*. Namely,

*x*_LE_(*t*) = *x*_LE_(θ_a_(*t*)) and *y*_LE_(*t*) = *y*_LE_(θ_a_(*t*)): horizontal and vertical positions of CoM of LE link

*x*_HAT_(*t*) = *x*_HAT_(θ(*t*)) and *y*_HAT_(*t*) = *y*_HAT_(θ(*t*)): those of HAT link

$x_{\text{CoM}}\left(t\right)=\frac{m_{\text{LE}}x_{\text{LE}}\left(t\right)+m_{\text{HAT}}x_{\text{HAT}}\left(t\right)}{m_{\text{LE}}+m_{\text{HAT}}}$ and $y_{\text{CoM}}\left(t\right)=\frac{m_{\text{LE}}y_{\text{LE}}\left(t\right)+m_{\text{HAT}}y_{\text{HAT}}\left(t\right)}{m_{\text{LE}}+m_{\text{HAT}}}$: those of the total CoM which can be compared with the corresponding experimentally obtained quantities.

For designing the active feedback controller, we consider a type of DIP-equivalent single inverted pendulum using a tilt angle θ_CoM_(*t*) of the total CoM at time *t*, which is defined as

$$\theta_{\text{CoM}}\left(t\right)=\tan^{-1}\left(\frac{x_{\text{CoM}}\left(t\right)}{y_{\text{CoM}}\left(t\right)}\right),$$

(see Supplementary Sections 1, 3 of Supplementary Materials). The intermittent control model hypothesizes that the supraspinal circuitry monitors the time-delay affected (quasi) state point ${\left(\theta_{\text{CoM}}\left(t-\Delta \right),{\dot{\theta }}_{\text{CoM}}\left(t-\Delta \right)\right)}^{T}$ on the phase plane of $\theta_{\text{CoM}}-{\dot{\theta }}_{\text{CoM}}$, where ${\dot{\theta }}_{\text{CoM}}$ is the velocity of θ_CoM_. According to the intermittent control model, the supraspinal circuitry determines the active ankle joint torque $\tau_{\text{a}}^{\text{act}}$ of *T*^act^ that switches between zero (OFF) and non-zero (ON) values depending on the location of the delay-affected state point as follows:

(3) $$\begin{array}{c} \tau_{\text{a}}^{\text{act}}\left(t\right)=\\ \left\{ \begin{array}{cc} 0 & \text{if}{\left(\theta_{.\mathrm{CoM}}\left(t-\Delta \right),{\dot{\theta }}_{\text{CoM}}\left(t-\Delta \right)\right)}^{T}\in {𝒟}_{\text{OFF}}\\ P\theta_{\text{CoM}}\left(t-\Delta \right)+D{\dot{\theta }}_{\text{CoM}}\left(t-\Delta \right) & \text{if}{\left(\theta_{\text{CoM}}\left(t-\Delta \right),{\dot{\theta }}_{\text{CoM}}\left(t-\Delta \right)\right)}^{T}\in {𝒟}_{\text{ON}} \end{array}\right. \end{array}$$

**FIGURE 1:**
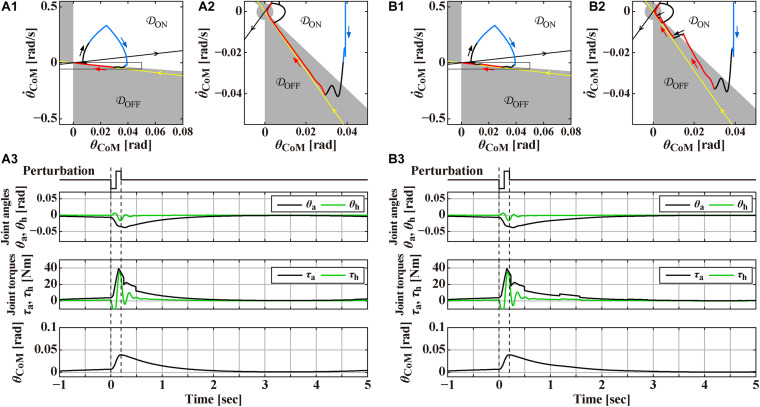
Responses to the support-surface perturbation of the double inverted pendulum model stabilized by an intermittent feedback controller and a reflexive control that operates transiently only for a short period of time after the perturbation at *t* = 0 s, to be compared with Figure 3 for the human experiment. The reflexive control is terminated at *t* = 0.25 s in panel **(A)** and *t* = 0.23 s in panel **(B)**. Overall structure of the response to the perturbation can be grasped by a rounded triangular trajectory of the state point in the $\theta_{\text{CoM}}-{\dot{\theta }}_{\text{CoM}}$ phase plane of the panels **(A1,B1)**, where the white (𝒟_ON_) and gray (𝒟_OFF_) areas in the $\theta_{\text{CoM}}-{\dot{\theta }}_{\text{CoM}}$ phase plane represents the ON and OFF regions of the intermittent control. Panels **(A2,B2)** are the magnifications of the squared areas of **(A1,B1)**, respectively. Black and red segments of the triangular trajectory are for the system, respectively, with the active feedback control (switched ON) and without the active feedback control (switched OFF). Blue segment of the trajectory is for the system with the reflexive control. Straight-shaped yellow and black lines, directing to and departing from the origin, respectively, represent the stable and unstable manifolds of the system in the absence of (i.e., switched OFF) active feedback control. Panels **(A3,B3)** are the time profiles of the ankle joint angle θ_a_ and the hip joint angle θ_h_, the corresponding joint torques, and the tilt angle θ_CoM_ for the total body CoM in response to the perturbation. See text for details.

**FIGURE 2:**
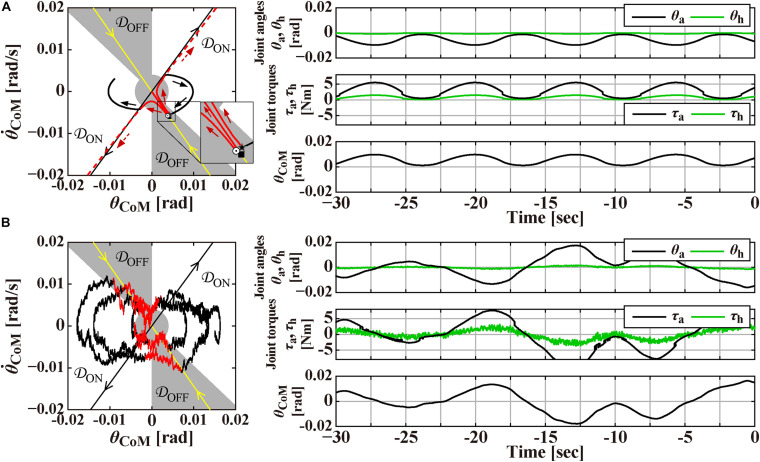
Magnification of Figure 1 around the origin for the time interval of [–30, 0] s, prior to the perturbation-onset at *t* = 0 s. One of the remarkable features of the intermittent control model is that the postural state point ${\left(\theta_{\text{CoM}},{\dot{\theta }}_{\text{CoM}}\right)}^{T}$ is not in a quiescent state even during quiet stance in the absence of noise as in panel **(A)**, but it is oscillating periodically with a small amplitude of tilt angle about 0.01 rad, as confirmed by a closed trajectory (limit cycle) on the $\theta_{\text{CoM}}-{\dot{\theta }}_{\text{CoM}}$ plane. Panel **(B)** is the case with small Gaussian white noise as a motor noise. Panels on the right-hand side of the phase planes are the corresponding waveforms of θ_a_ and θ_h_, the corresponding joint torques, and the tilt angle θ_CoM_. See text for details.

That is, $\tau_{\text{a}}^{\text{act}}$ is switched OFF, if the delay affected state point is located in the OFF-region denoted by 𝒟_OFF_ (see Figures 1, 2). It is switched ON, and operates according to the proportional (P) and derivative (D) feedback controller (delayed PD feedback controller), if the delay affected state point is located in the ON-region denoted by 𝒟_ON_. Theoretically speaking, the intermittency, i.e., switching between ON and OFF for the delayed feedback controller, is beneficial for avoiding the so-called delay induced instability in the feedback control systems, making the ankle joint flexible, and reducing mechanical energy consumption (Nomura et al., 2020).

We simulate dynamics of the model using a simple forward Euler integration without noise, i.e., *T*^n^ = 0 for most cases, but later with noise for making simulated dynamics more realistic. A perturbation is applied at *t* = 0 s, where the support-surface is shifted horizontally backward with an acceleration of α = −4.0 m/s^2^ for a duration of 100 ms, and then with α = 4.0 m/s^2^ (deceleration) for the subsequent 100 ms. We set an initial condition at (θ_*a*_,θ_*h*_)^*T*^ = (0.01,0)^*T*^ at time *t* = −50 s, far prior to the perturbation.

### Model-Simulated Quiet Stance and Postural Responses to the Perturbation

Figures 1A,B exemplify numerically simulated behaviors of the model for two slightly different conditions. In both cases, the DIP is in steady state of the quiet stance when the perturbation is applied at *t* = 0 s. The perturbation induces a large forward tilt (θ_CoM_ > 0) of the pendulum, followed by a process of postural recovery back to the upright equilibrium in a few seconds. Simulated postural dynamics are illustrated by waveforms of the joint angles θ_a_ and *θ_h_*, the total joint torques (τ_a_, τ_h_) ≡ *T*^pass^ + *T*^act^ + *T*^reflex^, the tilt angle θ_CoM_, as well as a trajectory of postural state point in the $\theta_{\text{CoM}}-{\dot{\theta }}_{\text{CoM}}$ phase plane.

The intermittent control model has been well characterized by its steady-state dynamics during quiet stance (Bottaro et al., 2008; Asai et al., 2009; Nomura et al., 2020; Suzuki et al., 2020). Figure 2A is a magnification of postural dynamics shown in Figure 1 for the time interval of [−30, 0] s prior to the perturbation-onset at *t* = 0 s. One of the remarkable features of the intermittent control model is that the postural state point ${\left(\theta_{\text{CoM}},{\dot{\theta }}_{\text{CoM}}\right)}^{T}$ is not in a quiescent state even during quiet stance in the absence of noise, but it is oscillating periodically with a small amplitude of tilt angle about 0.01 rad, as confirmed by a closed trajectory (limit cycle) on the $\theta_{\text{CoM}}-{\dot{\theta }}_{\text{CoM}}$ plane. In the intermittent control model, this oscillation corresponds to a noiseless version of postural sway during quiet stance. More importantly, the state point on the limit cycle is approaching the upright position (the origin of the $\theta_{\text{CoM}}-{\dot{\theta }}_{\text{CoM}}$ plane), though transiently, when it is located in the OFF-region, where the active feedback control is absent (switched OFF). Noting that the upright posture of the pendulum is unstable without active feedback control, getting close to the upright position during the OFF-period of the active feedback control might sound unintuitive. However, it is indeed a natural consequence of elementary physics. That is, a state point ${\left(\theta_{\text{CoM}}^{\text{⋆}},{\dot{\theta }}_{\text{CoM}}^{\text{⋆}}\right)}^{T}$ at the beginning of the OFF-period (marked by the star “⋆” on the limit cycle in Figure 2A) with a forward-tilted $\theta_{\text{CoM}}^{\text{⋆}}\;\left(>0\right)$ and a backward velocity ${\dot{\theta }}_{\text{CoM}}^{\text{⋆}}\;\left(<0\right)$ rotates backward around the ankle pivot by the law of inertia, against the gravitational toppling torque, with no help of the active control torque. The intermittent control model claims that the human central nervous system exploits such convergent dynamics of the non-actively controlled pendulum for stabilizing unstable upright posture. If the velocity ${\dot{\theta }}_{\text{CoM}}^{\text{⋆}}$ is small, the pendulum would eventually lose its kinetic energy, stop the motion, and then change the rotating direction to start falling forward, away from the upright position. This is the case of the limit cycle trajectory in Figure 2A. On the other hand, if the velocity ${\dot{\theta }}_{\text{CoM}}^{\text{⊙}}$ is large as shown for a state point ${\left(\theta_{\text{CoM}}^{\text{⊙}},{\dot{\theta }}_{\text{CoM}}^{\text{⊙}}\right)}^{T}$, marked by the symbol “⊙” in Figure 2A, the pendulum would reach the upright position, and pass by the upright position, and then fall backward. It is obvious that, for a given forward-tilt angle $\theta_{\text{CoM}}^{\text{■}}$, there exists a special velocity ${\dot{\theta }}_{\text{CoM}}^{\text{■}}$ (marked by the symbol “■” in Figure 2A), with which the pendulum falls neither forward nor backward, but it reaches slowly at the upright equilibrium point and stay there, despite the instability of the upright equilibrium point. The linear-shaped trajectory converging to the upright equilibrium point at the origin of the phase plane, i.e., a set of state points ${\left(\theta_{\text{CoM}}^{\text{■}},{\dot{\theta }}_{\text{CoM}}^{\text{■}}\right)}^{T}$ that reach the upright equilibrium point is called “the stable manifold” (the yellow line in Figure 2A) acting as a separatrix that determines whether the pendulum falls forward or backward, after getting close to the upright position. Because of this property, the uptight equilibrium point with no active feedback control is topologically classified as the “saddle,” representing an intersection point between crest and chine. In this way, the intermittent control model exploits the mechanical property of the inverted pendulum, for stabilizing unstable upright posture, such that the postural state near the stable manifold approaches the saddle-type upright equilibrium point transiently in the absence of active feedback control.

In Figure 2A, the state point on the limit cycle, which approaches the saddle point along the stable manifold, begins to fall forward eventually. Then, the state point enters the ON-region at the 1st quadrant of the phase plane, and the PD feedback control is switched ON, Δ seconds after the ${\left(\theta_{\text{CoM}}\left(t\right),{\dot{\theta }}_{\text{CoM}}\left(t\right)\right)}^{T}$ enters the ON-region. Note that, if the PD feedback is not switched ON, the pendulum falls forward continuously, as shown by the dashed trajectory in Figure 2A. Another important feature of the intermittent control is that the gains of the PD feedback controller (*P* and *D* values in Eq. 3) are much smaller, compared to the traditional postural control model (Maurer and Peterka, 2005), such that the PD feedback controller, even if it operates persistently, cannot stabilize the upright equilibrium (Nomura et al., 2013). Because of the small *P* and *D* values, the state point during the ON-period of the PD feedback controller does not get closer to the upright equilibrium at the origin, i.e., the PD feedback controller does not serve as a stabilizer. Instead, the state point rotates clockwise on the phase plane, moving from the 1st quadrant to the 4th quadrant, and returns to the OFF-region, where the PD feedback controller is switched OFF. Then, the state point approaches the upright equilibrium once again along the stable manifold, generating the limit cycle oscillation. Figure 2B is the exactly same process as Figure 2A, but with small additive white Gaussian noise *T*^n^, in which stochastic fluctuation of the state point mimics postural sway during quiet stance.

Now, we turn back to Figure 1 to look at the response to the perturbation. We assume for simplicity that the reflexive control also operates only at the ankle joint, which is defined as

(4) $$\tau_{\text{a}}^{\text{reflex}}\left(t\right)=P^{\text{reflex}}\theta_{\text{CoM}}\left(t-\Delta_{\text{reflex}}\right)+D^{\text{reflex}}{\dot{\theta }}_{\text{CoM}}\left(t-\Delta_{\text{reflex}}\right)$$

where Δ_reflex_ = 50ms is another feedback time-delay. Δ_*reflex*_ is shorter than Δ, in consideration of stereotypical nature of the spinal and supraspinal reflex arcs. We assume that the reflexive control operates transiently only for a short period of time (onset at *t* = Δ_reflex_ = 50ms, and offset at *t* = 250 ms in Figure 1A or *t* = 230 ms in Figure 1B) in response to the perturbation. Moreover, we assume large gains of the reflexive controller (*P*^reflex^ = 327 Nm/rad and *D*^reflex^ = 50 Nms/rad). Note that the intermittent PD feedback control $\tau_{\text{a}}^{\text{act}}$ is not operated when the reflexive control is in action. Note also that validation of the reflexive control is out of scope of this study, i.e., we do not intend to validate the model of reflexive feedback control quantitatively, although the current modeling would roughly be consistent with a recent report on a similar modeling on the postural control qualitatively (Zelei et al., 2021).

Overall structure of the response to the perturbation can be grasped by a rounded triangular trajectory of the state point in the $\theta_{\text{CoM}}-{\dot{\theta }}_{\text{CoM}}$ plane of Figure 1A-1. Note that a small knot-like portion on the left-bottom apex of the triangle represents the limit cycle oscillation during quiet stance before the perturbation. At the onset of the perturbation, the pendulum starts to tilt forward, and the tilting velocity increases rapidly, making part of the one side of the triangle (the thick black trajectory in Figure 1A-1). After Δ_reflex_ = 50ms from the perturbation-onset, the reflexive controller starts to operate, where the perturbed trajectory becomes blue in Figure 1A-1. At the top apex of the triangle (*t* = 100 ms), acceleration of the support-surface changes from α = −4.0 m/s^2^ to α = 4.0 m/s^2^, which reduces the forward-falling velocity of the pendulum.

The most notable postural recovery process in the model starts after the reflexive control is terminated. In the case of Figure 1A, the reflexive control is terminated at *t* = 250 ms, at which the delay-affected state point is still located in the ON-region. In Figure 1A-2, which magnifies a squared area of Figure 1A-1, the color of trajectory changes from blue (with the reflexive control) to black (with the intermittent PD feedback control in the ON-period). The state point with the intermittent PD feedback control in the ON-period keeps moving downward in the ON-region of 4th quadrant as in the case of quiet stance. Then, the state point enters the OFF-region, by which the intermittent PD feedback controller is switched OFF with the delay of Δ = 200 ms. Because the state point at the switch-OFF is located near the stable manifold, it approaches the upright equilibrium (the limit cycle, indeed) slowly along the stable manifold.

Physiological interpretation on the sensorimotor information processing performed by the central nervous system during this period is the major concern of this section. One might think that no sensory information about *θ_CoM_* and ${\dot{\theta }}_{\text{CoM}}$ is utilized for generating null active feedback torque ($\tau_{\text{a}}^{\text{act}}=0$) during the OFF-period of the intermittent controller. However, that would be wrong, because the central nervous system must monitor and pay attention continuously to determine whether the active control can remain switched OFF ongoingly, or it should be switched ON. Indeed, in the case if the reflexive control is terminated at *t* = 230 ms as in Figure 1B, which is slightly earlier than the case of Figure 1A, the intermittent controller shows a chattering-like ON-OFF switching after the state point enters the OFF-region that makes the intermittent controller switched OFF as in Figure 1A. In this case, as shown in Figure 1B-2, the state point that enters the OFF-region moves to the equilibrium point within the OFF-region along the stable manifold for about a second as in Figure 1A-2. However, it reaches and crosses the ON-OFF boundary that switches the PD controller on. Then, the state point moves downward according to the PD controller, and crosses the ON-OFF boundary back to the OFF-region, which switches the PD controller OFF, shortly after the preceding switch-ON event. A similar process repeats near the limit cycle, generating the chattering-like ON-OFF switching. In this way, the chattering-like ON-OFF switching at the ON-OFF boundary can be considered as an externalization of the underlying sensorimotor information processing, which is not externalized in the case of Figure 1A.

In summary, slow dynamics at the late phase of postural recovery along the stable manifold might be accompanied by the sensorimotor information processing with a continuous attention by the central nervous system to determine whether the active control can remain switched OFF ongoingly, or the active control should be switched ON, either with or without the chattering-like ON-OFF switching. This process to determine ON or OFF for the brain might be very similar to a decision-making process in Go-NoGo tasks, although ON-OFF selection is an automatic process, whereas Go-NoGo is a voluntary process. Because it has been known that Go-NoGo tasks are accompanied by the desynchronization of beta-band oscillation as well as the synchronization of beta-band oscillation (beta rebound), both for Go and NoGo responses (Alegre et al., 2004; Zhang et al., 2008; Swann et al., 2009), we expect that the sensorimotor information processing at the late phase of postural recovery along the stable manifold might also be accompanied by a similar brain activity. This is a model-based motive for exploring the EEG beta rebound in a time span of a few seconds after perturbing quiet stance.

Finally, panels below the $\theta_{\text{CoM}}-{\dot{\theta }}_{\text{CoM}}$ planes in Figures 1A,B, and those on the right-hand side of the *$\theta_{\text{CoM}}-{\dot{\theta }}_{\text{CoM}}$* planes in Figures 2A,B represent the waveforms of the joint angles (θ_a_ and θ_h_), the total joint torques (τ_a_,τ_h_)^*T*^≡*T*^pass^ + *T*^act^ + *T*^reflex^, and the tilt angle θ_CoM_. Those waveforms would be compared qualitatively with experimentally obtained postural responses.

## Materials and Methods

### Participants

Nine healthy young male participants (mean age 24.2 years, SD 1.4 years) were included in the study. None of the participants suffered from neurological disorders nor used medications that could influence posture. All participants gave written informed consent, which was executed in accordance with the principles of the Declaration of Helsinki (2013) and approved by the ethical committee of the Graduate School of Engineering Science at Osaka University.

### Experimental Protocol

Standing posture, electromyography (EMG), and EEG signals were measured under the following two conditions: quiet standing (control) and support-surface perturbation (perturbed). Postural sway during quiet stance in the control condition was measured to determine the equilibrium posture with accompanied EEG signals (baseline EEG) to be compared with postural recovery process, particularly in the period a few seconds prior to each perturbation. In each control or perturbed condition trial, participants were asked to stand still for 7 min on a treadmill (Bertec, Columbus, OH, United States) with their arms folded on their chest, while keeping their gaze fixed on a target located 4.5 m in front of them at the eye level. Specifically, the anterior-posterior (AP) direction of postural sway was defined to be parallel to the longitudinal direction of the treadmill belt. Although 7 min trials are relatively long, it has previously been shown that important sway metrics, including diffusion coefficients of the stabilogram at long- and short-term regimes, are not affected by long standing durations, suggesting that cortical activities associated with postural control are also not affected by long standing durations (e.g., van der Kooij et al., 2011). The participants performed two trials for each of the control and perturbed conditions (four 7 min trials in total). The order of four trials was chosen randomly from C-P-C-P, C-P-P-C, P-C-P-C, and P-C-C-P with equal probability, where C and P represent the control and the perturbed trials, respectively. A rest of at least 3 min was given between trials. During the control trials, the participants were asked to remain relaxed and still. During the perturbed trials, the support-surface perturbation was applied during upright stance by moving the treadmill-belt backward slightly and quickly using an in-house computer-program. Specifically, each perturbation spanned 200 ms, which was composed of an acceleration phase (α = −4.0 m/s^2^ for 100 ms), followed by a deceleration phase (α = 4.0 m/s^2^ for 100 ms). In each 7 min trial, 20 perturbations were applied with a fixed interval of 20 s between each perturbation. All participants could maintain upright stance against each perturbation without having to initiate compensatory steps or other overt movements in any of the trails. In all trails, participants were instructed to stand upright in a relaxed state to reduce the effects of muscle-activity-derived artifacts in EEG recordings.

### Experimental Setup

Postural kinematics were measured using a three-dimensional optical motion capture system (SMART-DX, BTS Bioengineering, Milan, Italy) with a sampling frequency of 300 Hz, where light reflection markers were attached on ankles, greater trochanters, and acromions of the left and right sides of the body. These markers were used to estimate joint angles and position of the total body CoM. The postural kinematics in the AP and medio-lateral (ML) directions were quantified by measuring the center of pressure (CoP), for which a force plate built in the treadmill acquired time-profiles of the ground reaction force vectors at a sampling frequency of 1,200 Hz. EMG signals were recorded from the ankle muscles of both the left and the right legs, including the soleus (SO), medial-gastrocnemius (MG) and tibialis anterior (TA) muscles. EMG data were recorded using wireless surface electromyograms (EMGs) (FreeEMG, BTS Bioengineering, Milan, Italy) with a sampling frequency of 1,000 Hz. EEG signals were measured using a 32-channel mobile bio-amplifier that was placed inside a backpack and worn by the participants and a waveguard cap that included active shielded cables for reducing movement-induced interference (eegosports, ANT Neuro, Hengelo, Netherlands). Specifically, Ag/AgCl electrodes were arranged in accordance to the International 10/10 system (Chatrian et al., 1985; Jurcak et al., 2007). All electrodes used CPz as a reference, and one frontal electrode was used as the ground (GND). The impedance in all electrodes was controlled to be less than 20 kΩ during the measurements (Ferree et al., 2001). All EEG signals were sampled at a sampling frequency of 2,048 Hz and stored on a computer for post-processing. The motion capture system (SMART-DX) and the EMG system were provided by the same vendor, in which A/D conversions were performed synchronously using a stroboscope-related clock. The CoP signals from the treadmill were also recorded synchronously by SMART-DX using the same A/D converter for the motion capture. Data sampling by the SMART-DX and the EEG systems were started simultaneously by the same start-trigger. Moreover, perturbation-event-makers generated by the SMART-DX were recorded into the EEG recording system, by which recording data of the SMART-DX and the EEG system were aligned at every perturbation-onset.

### CoP Analysis During Quiet Stance

Postural sway data during quiet stance (control condition) were characterized using the CoP time-series, which were mainly used for manifesting postural responses to the perturbations in comparison with postural sway during quiet standing. In short, the following parameters were computed: (i) standard deviations of CoP fluctuations in the AP and ML directions; and (ii) slopes of linear regression lines for the log-log plotted power spectrum of CoP in the AP direction at low (0.02–0.2 Hz) and high (1–8 Hz) frequency regimes, as described elsewhere (Yamamoto et al., 2011, 2015; Matsuda et al., 2016). Particularly, it is known that the power spectrum in the low frequency regime exhibits the *f*^–β^-type scaling with the exponent close to β = 1.5, which is one of the hallmarks of the intermittent control hypothesis (Collins and De Luca, 1994; Asai et al., 2009; Nomura et al., 2013; Yamamoto et al., 2015). Parameters defined here were computed from the entire 7 min CoP time-series and the results obtained on the two control trials were averaged to obtain the CoP postural sway measures for each participant.

### Event-Locked Average During Perturbed Stance

**FIGURE 3:**
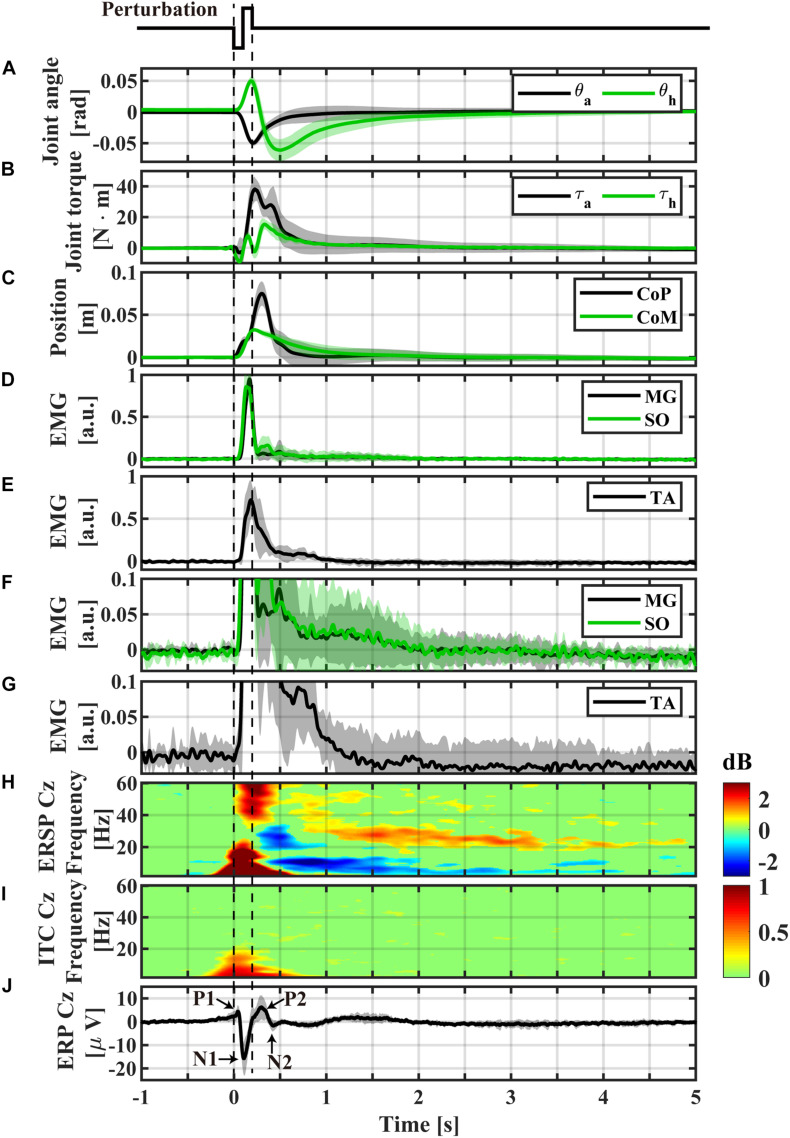
Event-locked average profiles (averaged across participants) triggered by the perturbation-onset. **(A)** Ankle and hip joint angles, **(B)** joint torques obtained by the inverse dynamics analysis, **(C)** CoP and CoM positions, **(D)** normalized EMGs of Medial-Gastrocnemius and Soleus, **(E)** normalized EMG of Tibialis Anterior, **(F)** magnification of panel **(D)**, **(G)** magnification of panel **(E)**, **(H)** ERSP of Cz electrode, **(I)** ITC of Cz electrode, **(J)** ERP of Cz electrode. The light color shaded area in each of panel **(A–G,J)** is the standard deviation, representing the distribution across participants. Non-significant differences from baseline (bootstrap statistics, *p* > 0.05) were set to 0 dB and colored by green in panels **(H,I)**. Powers for areas with red and blue (non-green) colors were significantly larger (i.e., ERS) and smaller (i.e., ERD) than the baseline power, respectively.

For the perturbed condition, postural responses were characterized with respect to the perturbation-onset by event-locked average profiles of the ankle and hip joint angles as well as the position and velocity of CoM and CoP for each participant. For the event-locked average, time-series data were segmented into many small pieces of the data of 20 s long, referred to as epochs, each of which is a response to a single perturbation from −5 to 15 s. Specifically, epochs from the two perturbed trails were pulled together, resulting in 40 perturbations (40 epochs) for each participant. Event-locked average time-profile was obtained by calculating the mean of the 40 epochs for each participant, where the onset of each perturbation was used as a triggering event. In addition to the participant-wise averaging, averaged profiles across participants were also computed (Figure 3). The event-locked average joint angles, CoM and CoP profiles were compared with the corresponding time-profiles of event-locked average responses of EMGs, ERPs and ERSPs. For our analysis, each time-profile of joint angle, joint torque, CoM, CoP, EMGs, and ERPs of EEG was plotted after subtracting its mean. Moreover, the maximum and/or the second maximum peaks of the event-locked average responses in positive and/or negative directions were detected, for which latencies from the triggering event were obtained.

### Estimation of Joint Angles and CoM for Perturbed Stance

Joint angles and position of the total body CoM were estimated only for the perturbed stance. To this end, the triple-inverted pendulum, defined in section “Theoretical Background” (with Supplementary Figure 1 and Supplementary Sections 1–3 of Supplementary Materials), with Foot, LE and HAT links was used, which is essentially DIP with the spatially fixed Foot link. The ankle joint angle θ_a_ and the hip joint angle θ_h_ were defined in the same way as in section “Theoretical Background.” Plantar flexion for the ankle joint and extension for the hip joint were defined as the positive direction. θ_a_ and θ_h_ for each participant were estimated using the motion-captured positions of the markers in the global coordinate system. Specifically, the positions of two corresponding markers on the left and right sides of the body were projected on the sagittal plane, and then averaged to obtain the position of each of the ankle, greater trochanter, and acromion in the model. For full details related to the model and joint angles estimation, see Supplementary Sections 1, 2 in Supplementary Materials.

Horizontal position of the total CoM in the AP direction during standing was estimated from the CoM-positions and the masses of the HAT link (*m*_HAT_) and the LE link (*m*_LE_) of the model as described in section “Theoretical Background.” *m*_HAT_ and *m*_LE_ were estimated from the total body weight of each participant using a statistical formula of *m*_HAT_:*m*_LE_ = 0.62:0.35 (Suzuki et al., 2012). We assumed that the CoM of each link was located at the middle point of the link. CoM time-series were smoothed using a zero-lag fourth-order Butterworth filter with a cut-off frequency of 10 Hz. CoM velocity time-series were calculated using the central difference method for the filtered CoM data. Note that the tilt angle of the total CoM is represented by θ_CoM_ for the model used in section “Theoretical Background.” For full details related to CoM estimation, see Supplementary Section 3 of Supplementary Materials.

### CoP Analysis During Perturbed Stance

In the perturbed condition, the foot position shifted backward in response to every perturbation due to the backward translation of the support surface (i.e., the treadmill belt). Because the built-in force plate and its local coordinate system were fixed in the global coordinate system independent of the moving belt, we obtained the time-series of CoP positions relative to the foot, which represents the actual postural response to the perturbation, by subtracting ankle position (that moved together with the belt) from measured CoP time-series. For full details of the CoP processing, see Supplementary Section 4 of Supplementary Materials. The CoP time-series was then low-pass filtered in post-processing using a zero-lag fourth-order Butterworth filter with a cut-off frequency of 10 Hz, while the CoP velocity time-series was obtained using the central difference method for the filtered CoP data.

**FIGURE 4:**
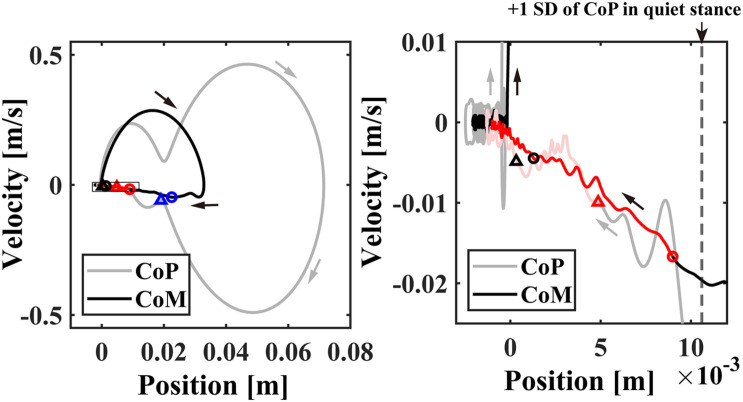
The event-locked average of CoP/CoM profile (averaged across subjects) in the CoP/CoM-position vs. the CoP/CoM-velocity phase plane for –3 < *t* < 8 s. The small blue, red, and black circles plotted on the CoM (black line) trajectories in the left and/or right panels indicate the time instants of 0.5, 1.0, and 2.0 s, respectively. The triangles with the same colors indicate the corresponding time instants on the CoP (gray line) trajectories. The right panel is an enlargement of the rectangular region near the origin in the left panel. The time-duration with beta rebound (ERS) about 1.0 < *t* < 4.0 s with significantly large power was indicated by the red trajectories. The vertical dashed line in the right panel indicates the standard deviation of the postural sway during the quiet standing.

To obtain measures of postural stability throughout the duration of the perturbation-induced postural responses, the event-locked average CoP and CoM responses averaged across participants were plotted as a function of time (Figure 3C). Moreover, they were plotted on the CoP/CoM-position vs. CoP/CoM-velocity plane (Figure 4) as a phase plane to compare dynamics of the responses with dynamics of the model described in section “Theoretical Background” and in previous studies (Bottaro et al., 2005, 2008; Asai et al., 2009).

### Joint Torque Analysis During Perturbed Stance

Joint torques exerted on the ankle τ_a_ and the hip τ_h_ as the sum of passive and active torques during postural recovery responses, corresponding to (τ_a_,τ_h_)^*T*^≡*T*^pass^ + *T*^act^ + *T*^reflex^ defined in section “Theoretical Background,” were estimated by solving inverse dynamics based on the triple-inverted pendulum model with the Foot link fixed on the moving support-surface, the motion captured body kinematics (θ_a_ and θ_h_), the CoP positions, and the ground reaction force vectors. For full details related to the inverse-dynamics analysis, see Supplementary Section 5 of Supplementary Materials.

### EMG Analysis

The EMG data recorded from MG, SO, and TA muscles were processed using a zero-lag 20–450 Hz band-pass fourth-order Butterworth filter, full-wave rectified, and then low-pass filtered using a zero-lag second-order Butterworth filter with a cut-off frequency of 15 Hz (Merletti and Di Torino, 1999; Yoshida et al., 2017). The processed EMGs for each muscle from the left and right limbs were then averaged for each participant, because we were particularly interested in the postural dynamics in the AP-direction. Then, an event-locked average of EMG for each muscle was normalized by its maximum value using the peak of the EMG profile for each participant. The normalized event-locked average of EMGs were used to analyze peak latency for each muscle.

### EEG Analysis

Pre-processing, denoising, and analysis of EEG signals were conducted using EEGLAB (Delorme and Makeig, 2004; Loo et al., 2019). For full details about EEG pre-processing, see Supplementary Section 7 of Supplementary Materials. Here, we present a summary. First, EEG data were down-sampled to 1,000 Hz. A zero-lag high-pass first-order Butterworth filter with a cut-off frequency of 1 Hz was applied (Winkler et al., 2015). We then removed data from noisy electrodes whose correlation coefficients between the surrounding electrodes were smaller than 0.8 (Bigdely-Shamlo et al., 2015), and they were not used in the following analysis. The average number of electrodes rejected in single trials was 0.28 out of 32 electrodes. We performed the artifact subspace reconstruction (ASR), which is a method for denoising EEG signals (Mullen et al., 2015). After ASR, the removed data from the noisy electrodes according to the criteria described above were replaced by the data from the surrounding electrodes using linearly spatial interpolations (Bigdely-Shamlo et al., 2015). Then, re-referencing was performed based on the average potential of all the electrodes, by which possible undesirable effects caused by a specific choice of the reference electrode (CPz in this study), if any, was minimized. Independent component analysis was performed to remove the independent components (ICs) originated from EMG and electrooculograms (EOGs) activities using a method described by Bruijn et al. (2015). For single trials, the mean number of removed EMG components was 2.3, and the mean number of removed EOG components was 1.6, out of 32 ICs. After removing those artifact ICs, the remaining ICs were re-mapped onto the electrodes. The re-mapped EEG data in the time interval from 5 s prior to the perturbation until 15 s after the perturbation was then used as an epoch to analyze cortical activity. To confirm that no apparent contamination of EMG signals due to activity of the craniofacial muscles into EEG, power spectra of all electrodes were examined, particularly for 0–500 ms time interval after the perturbation, and confirmed monotonic decreasing shape, which is typical for EMG-noise-free EEG signals, in all spectra (Supplementary Figure 2).

**FIGURE 5:**
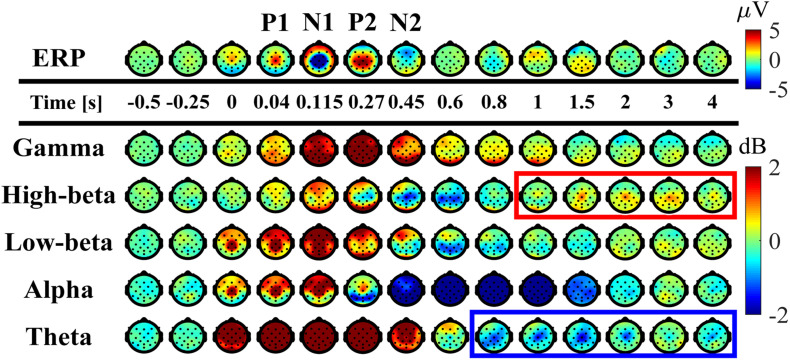
Spatial distributions of ERP and ERSP on the scalp (averaged across participants). Upper panel: time-changes in the spatial distribution of the potential on the scalp. Lower panels: time-changes in the spatial distribution of EEG-power, i.e., ERSP, for the frequency-bands of theta (4–7 Hz), alpha (8–13 Hz), low-beta (13–20 Hz), high-beta (21–30 Hz), gamma (40–60 Hz) plotted on the scalp. The scalps surrounded by the red and blue rectangles indicate that beta ERS whose amplitude was significantly larger than the mean power before perturbation and theta ERD which were significantly smaller, respectively, at the Cz electrode.

Event-related potential was calculated for each electrode by computing the event-locked average over all epochs (Davis et al., 1939; Quant et al., 2005). The largest and the second largest peak amplitudes in positive and negative directions for the ERP time-profile at Cz electrode were detected for each participant. Those peaks were expected to correspond to P1, N1, P2, and N2 potentials (Quant et al., 2005; Payne et al., 2018; Payne and Ting, 2020). Moreover, ERPs were plotted on the scalp as a function of time (snapshots) to characterize spatio-temporal distribution of the ERP responses (Figure 5).

Outcomes of time-frequency analysis using the wavelet transform for each epoch for each electrode were summarized by averaging over epochs for all perturbations, for each participant and across participants to obtain ERSPs (Makeig, 1993). For full details about the wavelet transform, see Supplementary Section 8 of Supplementary Materials. Particularly, ERSP for Cz electrode was presented by plotting wavelet coefficients as a function of time (*t*) and frequency (*f*) on the time-frequency plane. For this plot, frequency-wise averaged powers for 4 s in the interval [−5, −1] s prior to each perturbation was used as the baseline, and ERSP power for each frequency was represented in dB with respect to the corresponding baseline power. The significances of ERD/ERS responses were tested using a non-parametric bootstrapping method (Delorme and Makeig, 2004). The significance level was set at *p* < 0.05. As a result, areas in ERSP that were not significantly different from the baseline were set to 0 dB and colored by green in ERSP (Figure 3H). Areas that were significantly enhanced (ERS) and attenuated (ERD) were identified, and colored by red and blue, respectively. Moreover, inter-trial coherence (ITC) was calculated along with the estimation of ERSP to examine whether or not ERS and ERD are phased-locked (Figure 3I), in which the significance was verified with a significance level of *p* < 0.05.

**FIGURE 6:**
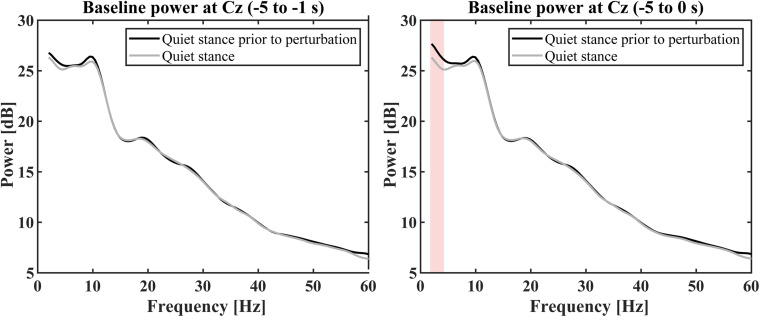
Grand average baseline power spectra in quiet stance prior to perturbation (black curve) and quiet stance (gray curve). The left panel shows the average power calculated from –5 to –1 s before the perturbation. There was no significant difference between the baseline of perturbed conditions and the quiet stance power spectrum (*p* < 0.05). The right panel shows the average power calculated from –5 to 0 s before the perturbation. There was no significant difference between the baseline in perturbation conditions and the quiet stance power spectrum, except for powers from 2 to 4.5 Hz (red color shaded area, *p* < 0.05).

For the ERSP estimation, baseline powers of Cz electrode for the perturbed condition, which was computed for the time interval of [−5, −1] s prior to each perturbation, were compared with the power spectrum during quiet stance (control condition) to examine whether the baseline powers were affected by the perturbation-induced response, i.e., to reject a possibility that EEG alters prior to the perturbation in a predictive manner (Figure 6). The comparisons for this examination were performed using Wilcoxon signed rank tests corrected using false discovery rate at a significance level of *p* < 0.05 (Benjamini and Hochberg, 1995) for selected frequency-bins from 2 to 60 Hz with a frequency resolution of 0.5 Hz. Because it is important to calculate the baseline power from an appropriate time (Pfurtscheller, 2006), although the time interval of [−5, −1] s for the baseline period was selected to avoid the effect of “power-leakage” at the interval immediate before the perturbation due to a poor temporal resolution of the wavelet at low frequency regime, frequency-wise averaged powers for the time interval of [−5, 0] s prior to each perturbation was also compared with the power spectrum during quiet stance (control condition) in the same way for [−5, −1] s.

Because an actual frequency band with ERD/ERS at a selected frequency band (such as theta, beta or gamma) is individual-dependent in general, one needs to adjust the lower (or upper) limit of the band frequency for ensemble average across individuals. However, it was not the case in the current study as confirmed by the data analyses for each of the nine participants, separately in Supplementary Figures 3–11 in Supplementary Section 9 of Supplementary Materials. That is, ERD/ERS at beta and theta bands that we analyzed in this study appeared at the frequency bands defined commonly across participants. For this reason, the ensemble average of ERSPs was performed by simple arithmetic means without tuning the frequency range. Moreover, the temporal change (snapshots) of the spatial distribution of power on the scalp using the average amplitude for each of the frequency bands at theta (4–7 Hz), alpha (8–12 Hz), low-beta (13–20 Hz), high-beta (21–30 Hz), and gamma (40–60 Hz) were plotted (Figure 5).

## Results

### CoP During Quiet Stance

Postural sway during quiet stance (control condition) can be characterized as follows. The average of CoP variability in AP direction was 10.6 ± 4.9 mm and that of in ML direction was 6.4 ± 3.8 mm. The slope of the linear regression line for the log-log plotted power spectrum of CoP in the AP direction at the low frequency regime was −1.68 ± 0.35, and that at the high frequency regime was −2.85 ± 0.48.

### Kinematic and Kinetic Responses to the Perturbations

Temporal patterns of kinematic and kinetic responses in Figure 3, averaged across participants, were qualitatively the same as simulated responses for the intermittent control model in section “Theoretical Background” (Figures 1A-3,B-3), although quantitative differences in amplitude of responses were apparent. In this sequel, temporal patterns of kinematic and kinetic responses were described briefly.

During support-surface acceleration phase (0 < *t* < 100 ms), the LE link tilted forward (θ_a_ in Figure 3A), i.e., the proximal end of the LE link moved forward, due to the sudden backward shift of the feet at the distal end of the LE link. The forward shift of the proximal end of the LE link induced a forward shift in the distal end of the HAT link, resulted in the backward tilt of the HAT link relative to the LE link (θ_h_ in Figure 3A). The anti-phase movement of the ankle and hip joint angles at the initial phase of the postural response resulted in the forward shift of CoM below 0.01 m (Figure 3C at *t* ∼ 100 ms).

During the support-surface deceleration (100 < *t* < 200 ms), inertial forces pulled the body backward, i.e., in the opposite direction to that during the acceleration period. However, they were not large enough to reverse the movement directions of θ_a_ and θ_h_. Thus, the LE link continued tilting forward, and the HAT link continued rotating backward relative to the LE link (Figure 3A for 100 < *t* < 200 ms).

In the middle of the acceleration period (*t* ∼ 60 ms), SO and MG activities were initiated, and peaked at *t* ∼ 150 ms in the middle of deceleration (Figure 3D). Those muscle activities generated plantar-flexion torque at the ankle to brake the forward tilt (Figure 3B with the upward plantar-flexion peak of the ankle joint torque at *t* ∼ 210 ms), leading to the reverse in the rotation direction of θ_a_ from the negative/dorsiflexion direction to the plantarflexion direction at the downward dorsiflexion peak at *t* ∼ 200 ms in Figure 3A. At around the same time, TA activity reached at the peak (Figure 3E).

The direction reversal in the LE link (θ_a_) toward the upright position at *t* ∼ 200 ms initiated the backward shift of the distal end of the HAT link, which induced the reversal in θ_h_ to the negative/flexion direction (the upward peak of the hip joint extension at *t* ∼ 200 ms in Figure 3A). A contribution of the hip joint torque for this direction reversal was small, as confirmed by the small hip joint torque at *t* ∼ 200 ms in Figure 3B.

The downward dorsiflexion peak of θ_a_ coincided with the peak of the CoM forward-shift at *t* ∼ 200 ms in Figure 3C. The CoP continued to move forward after the CoM peak, and peaked at *t*∼ 300 ms.

After the dorsiflexion peak, θ_a_ decayed monotonously, and almost recovered the equilibrium at *t* ∼ 1.0 s. The corresponding ankle joint torque decreased to almost zero at *t* ∼ 2.0 s. The hip joint angle θ_h_ decreased in the negative/flexion direction until it reached the downward hip-flexion peak at *t* ∼ 500 ms, reversed again to the positive/extension direction (Figure 3A). In contrast to the hip-extension peak, the direction reversal at the hip-flexion peak was induced by the hip joint torque peaked at *t* ∼ 330 ms in Figure 3B. After the hip-flexion peak, θ_h_ and the hip joint torque decayed monotonously to the equilibrium within the interval of 2.0 < *t* < 3.0 s, which should be compared with the EEG activity lasting longer than this recovery period as shown later.

### Phase Plane Trajectories

Event-locked average of CoP/CoM trajectories (averaged across participants) in the CoP/CoM-position vs. the CoP/CoM-velocity phase plane are shown in Figure 4, which can be compared with Figure 1 for the intermittent control model. As in Figures 1A,B, the left panel of Figure 4 spans the range of whole responses that exhibit perturbation-induced large excursions, whereas the right panel spans a range of postural sway during quiet stance to display the CoP/CoM responses at the very late phase of the postural recovery. Similar to Figure 1 for the intermittent control model, both of CoM and CoP trajectories after *t* = 1.0 s (the red circles and triangles) approached the equilibrium posture at the origin in a linear manner, i.e., each of CoP and CoM trajectories was line-shaped toward the origin with no oscillations around the origin. The postural state after *t* = 2.0 s (the black circles and triangles) was very close to the equilibrium, but still moving along the above-mentioned line, meaning that the postural state did not move randomly even within this small region near the origin. In other words, the postural recovery dynamics in this last phase still exhibited strong deterministic structure that were not vanished by the event-locked averaging.

### EEG Responses Revealed by ERP and ERSP

Figure 3H represents the ERSP and with the ITC for the Cz electrode (Figure 3I), while Figure 3J represents the ERP time-profiles. In Figure 5, the spatial distributions of ERP (top trace) and ERSP for five frequency-bands are plotted on the scalp as snapshots at several latencies for −0.5 < *t* < 4.0 s. It was confirmed by Figure 6 that there was no significant difference between the baseline powers of the ERSP (the powers for [−5, −1] s prior to the perturbation) and the powers during quiet stance for any frequencies (Figure 6-left). Moreover, the powers for [−5, 0] s were also not significantly different from the powers during quiet stance for any frequencies, except for a narrow frequency band between 2 and 4.5 Hz (Figure 6-right). For individual responses to the perturbation prior to the ensemble across participants, see Supplementary Figures 3–11 in Supplementary Section 9 of Supplementary Materials.

As reported in the previous studies (Quant et al., 2005; Payne et al., 2018; Payne and Ting, 2020), the ERPs of P1, N1, P2, and N2 for the early phase of the response were reconfirmed at the Cz electrode (Figures 3J, 5-top). The P1 was spatially localized around the Cz electrode (Figure 5 at 40 ms). For the Cz electrode, latency and peak amplitude of the P1 potential were 41 ± 18 ms and 5.1 ± 2.3 μV, respectively. The P1 potential was immediate before the onset of SO, MG, and TA activations. The N1 and P2 potentials also distributed around the Cz electrode (Figure 5 at 115 and 270 ms). Latency and peak amplitude of the N1 potential at the Cz electrode were 116 ± 22 ms and −17.2 ± 6.2 μV, respectively. The N1 potential was immediately after the onset of support-surface deceleration, and it was followed a few tens of milliseconds by the peaks of SO, MG, and TA activities (Figures 3D,E). Latency and peak amplitude of the P2 potential were 264 ± 49 ms and 8.5 ± 3.6 μV, respectively. The P2 response was a few tens of milliseconds after the termination of the support-surface deceleration, which was immediately after the end of large activations of SO, MG, in the middle of the large TA activation. Unlike the other potentials, the N2 potential was distributed around the Fz electrode located at the frontal midline (Figure 5 at 450 ms).

The ERS for the early phase of the response at the low-beta, alpha, theta, and gamma frequency-bands appeared within 300 ms from the perturbation-onset (Figures 3H, 5). The low-beta ERS peaked at 94 ± 20 ms, the alpha ERS peaked at 92 ± 26 ms, the theta ERS peaked at 124 ± 30 ms, and the gamma ERS peaked at 252 ± 106 ms for Cz (Figure 3H). Note that the ERS was absent specifically for the high-beta band. The low-beta ERS and theta ERS were spatially distributed over the scalp, but the alpha ERS was localized around Cz electrode (Figure 5). The ITC corresponding to the ERS at low-beta, alpha, and theta was significantly higher in the Cz electrode (Figure 3I). The gamma ERS was spatially distributed over the scalp, and it showed a particularly strong power in the occipital area, corresponding in time to the peak of forward-shift of CoP (Figure 3C).

For the late phase of postural recovery (300 < *t* < 600 ms), ERD at high-beta band (high-beta ERD) was observed dominantly at Cz (Figure 3H) and weakly at C3 and C4 (Figure 5). The peak latency of the high-beta ERD was 450 ± 83 ms for Cz (Figure 3H). Moreover, the high-beta ERD coincided with the time instant when the CoP on the way back to the upright position caught and surpassed the CoM that had preceded the CoP until this point (Figure 3C). Note that the positive and negative ankle torques represent, respectively, pulling the forward-tilted posture backward and braking the backward moving posture. This ankle torque was mostly generated by body mechanics, i.e., it was induced by the relative positioning between CoP and CoM, which could be confirmed by the fact that the large EMG responses of SO, MG, and TA were already in the late of their decreasing phases, although those muscle activities also contributed to the stabilization (Figures 3B,F,G). In this way, the high-beta ERD did not necessarily appear in the middle of the large EMG responses, but it appeared at the tail of the large responses of SO and MG. Since the large activation of TA was delayed relative to those of SO and MG (about 50 ms difference in the peak latencies), the high-beta ERD overlapped with the late phase of the large TA activation that brakes the backward recovery movement.

For latencies longer than 1.0 s, we showed two significant event-related clusters of powers in ERSP (Figure 3H), which were the main findings of this study. One was the ERS at high-beta band (high-beta ERS), which appeared after the high-beta ERD. The high-beta ERS sustained over a relatively long duration with its peak latency located at 3.27 ± 1.35 s. The high-beta ERS was observed mainly at Cz electrode (Figure 5). The averaged powers across participants at the high-beta band in the latency of 1 < *t* < 4 s were significantly greater than the resting potential (*p* < 0.05), i.e., the beta rebound sustained for as long as 3 s.

During the appearance of the high-beta ERS, the CoP and the CoM became close to each other in the very late phase of the recovery process (Figure 3C). This could also be confirmed by Figure 4 for the trajectories of postural state in the CoP/CoM-CoP/CoM velocity phase plane. Particularly, the trajectories within the period when the high-beta ERS was significantly large is indicated by the red curve segments in the phase plane (Figure 4-right), by which we could observe that the postural state during the appearance of the high-beta ERS was located within the range of postural sway during quiet stance. However, as described above, the postural state (CoP/CoM) accompanied by the high-beta ERS did not move randomly, but it moved deterministically along the above-mentioned line toward the origin. Comparison between ERSP in Figure 3H and enlarged EMGs in Figures 3F,G showed that activations of SO and MG muscles had become small compared to those of short latency (<0.3 s), but they were still slightly larger than those during quiet stance for the early period (1 < *t* < 2.5 s) of the high-beta ERS. However, activations of all muscles were the same as those during quiet stance for the later phase (2.5 < *t* < 4 s) of the high-beta ERS. That is, the high-beta ERS in its later phase was generated with no perturbation-induced excessive muscular activations.

In addition to the high-beta ERS, ERD at alpha and theta bands were also observed after the reflexive responses at early phase. As shown in Figure 5, the alpha ERD appeared at the occipital region for the early phase (*t* ∼ 270 ms), and then expanded to the whole head, with the latency 0.83 ± 0.17 s at Cz. Then, the theta ERD appeared specifically around Cz with its latency of 1.88 ± 0.74 s. The theta ERD, whose power was significantly smaller than the resting potential (*p* < 0.05), also exhibited a long-lasting property as in the high-beta ERS, as clearly confirmed in Figure 3H with the blue-colored ERD at theta band until the latency of 4.0 s, along with the red-colored ERS at high-beta band.

## Discussion

In this study, we examined the postural recovery process during upright stance in response to the support-surface impulsive perturbation. Slower time-scale of mechanical dynamics during postural recovery, compared to movements of the upper extremities, prompted our analysis of the temporal profile of cortical activations during postural stabilizing process. Particularly, based on preceding numerical simulations of the intermittent postural control model in section “Theoretical Background,” it was expected that the late phase of the postural recovery, which is characterized theoretically by slow dynamics along a stable manifold of the saddle-type unstable upright equilibrium point in the absence of active feedback control, might be accompanied with the sensorimotor information processing. In particular, such information processing requires continuous attention to the postural state by the central nervous system to determine whether the active control can remain switched OFF ongoingly, or the active control should be switched ON. We showed that neural responses for re-stabilizing upright posture lasted over a relatively long periods of time (i.e., few seconds), which is consistent to our hypothesis. Specifically, we identified a novel type of high-beta band cortical activity, i.e., high-beta ERD and subsequent high-beta ERS (beta rebound), as well as theta ERD, localized around Cz electrode over the primary motor area. Appearances of the beta ERD and ERS are basically consistent to the response that has been identified for motor tasks by the upper extremities, while the responses for the upper-limb tasks were much shorter and typically lasting only for a few 100 ms or 1 s in the longest case (Pfurtscheller et al., 1996; Pfurtscheller and Lopes da Silva, 1999; Engel and Fries, 2010). However, the temporal configuration of the beta ERS relative to the kinematic and kinetic responses of the perturbed stance was qualitatively different from those for the upper extremities. That is, the high-beta ERS for the perturbed stance started before the postural recovery dynamics, including EMG activities, were completed, and it was long-lasting for about 3 s along with a small residual motion during the postural recovery (Figures 3H, 4). This is in stark contrast to the beta ERS for the motor tasks with upper extremities that started after movements have been completely terminated.

Discussion below is organized as follows. First, in section “Responses in the Early Phase of the Perturbation,” before discussing our main findings on the high-beta ERS, which occurred in the later phase of the postural response, reflexive cortical responses during the early phase of the postural recovery process within about 300 ms after the perturbation-onset are summarized and discussed. Then, high-beta ERD and high-beta ERS (beta rebound), together with theta ERD, which occurred in the late phase of the response, are discussed in sections “Desynchronization of Cortical Activities at the End of the Initial Reflexive Phase” and “Long-Lasting High-Beta Rebound.” Particularly, possible mechanisms of long-lasting high-beta ERS and theta ERD were speculated based on our experimental findings in this study, in conjunction with theoretical aspects of the intermittent control model, together with limitations of the current study. In section “Neuroanatomical Interpretations of the Recovery Process,” we discuss a possible rationale for the spatial localization of long-lasting beta ERS and theta ERD at parietal cortex (Cz electrode), rather than frontal cortex, based on the most-advanced neuroanatomical model of the posture and gait control (Takakusaki, 2017), and finally we discuss limitations and future issues of this study.

### Responses in the Early Phase of the Perturbation

We confirmed P1, N1, and P2 peaks of the ERPs that distributed around Cz electrode (Figures 3H, 5), consistent with the previous studies (Quant et al., 2005; Payne et al., 2018; Payne and Ting, 2020). Following the perturbation, the first response P1 was observed around 41 ms. This delay is consistent with temporal profile of vestibular and somatosensory evoked potentials. Thus, P1 can be considered as the sensory recognition of the event. The short latency of P1 is also close to that of vestibular-evoked potential (Todd et al., 2014), which might generate the onset of EMGs of SO and MG as the vestibular-spinal reflex, earlier than 100 ms. Such descending signals to the spinal cord might be an origin of the fastest cortical modulations of spinal excitability during standing, which has been characterized previously by gains of H-reflex and TMS-based motor evoked potentials at the latency around 100 ms in response to a support-surface perturbation (Taube et al., 2006).

It has been considered that N1 represents neural processing of sensory information necessary for coordinating reactive balance responses (Dietz et al., 1984, 1985; Dimitrov et al., 1996; Staines et al., 2001; Quant et al., 2004; Payne et al., 2018; Payne and Ting, 2020). Moreover, it might represent an error signal for postural instability (Adkin et al., 2006, 2008; Mochizuki et al., 2009; Payne et al., 2019). A recent study reported that N1 amplitudes alter depending on postural balance functionality, i.e., larger N1 reported for individuals with lower balance ability, and smaller N1 responses for those with better balance (Payne and Ting, 2020). Taken together, these results imply that N1 expresses a supraspinal modulation of spinal reflexes. The latency of N1 responses for the reflexive reaction in the current study is highly consistent with temporal profile of information processing in simple reaction-time tasks (Donchin and Lindsley, 1966). That is, both in the current reflexive reaction and the simple reaction-time tasks, following the vestibular/somatosensory-evoked P1 response with the latency of about 50 ms (Cruccu et al., 2008), the fast vestibular-spinal reflex is generated, which modulates the spinal excitability (Taube et al., 2006). The vestibular and other sensory information might be processed for about 100 ms in the supraspinal circuitry, generating the N1 response with the latency of about 150 ms (Quinzi et al., 2019). Then, another 30–40 ms might be required for the neural transmission of descending signals to modulate the vestibular-spinal reflex for shaping the corrective responses. The sum of these time intervals yields about 200 ms, which is comparable with the peak activation latencies of the EMG responses in the current study (Figure 3D). Moreover, the current study showed that P2 was observed following the end of large muscle responses. Thus, the P2 can be interpreted as a re-afferent phenomenon. In this way, we could confirm typical ERPs with short latencies that have been examined so far during the perturbed stance. Existence of those ERPs, despite the differences in detailed experimental setup and protocol for various studies, indicates that they are robust and stereotypical. See section “Neuroanatomical Interpretations of the Recovery Process” for a neuroanatomical interpretation of the process in the early phase, together with the process in the late phase.

The ERS of theta, alpha, and low-beta bands were observed during perturbation-evoked N1. As reported in the previous studies (Varghese et al., 2014), these ERSs are phase-locked ERS, which can be confirmed by the ITC (Figure 3I), exhibiting significantly larger correlations compared to the baseline at the time-frequency regime corresponding to these ERS. A signal source for these ERS might be the sensorimotor cortex, as reported by Solis-Escalante et al. (2019). After the N1 response, the gamma ERS appeared between N1 and N2. Although the gamma ERS distributed over the whole head, it was the most prominent in the occipital area, representing a neural detection of postural instability (Slobounov et al., 2005). As suggested by Solis-Escalante et al. (2019), a major signal source of the Gamma ERS might be located at the occipital lobe.

### Desynchronization of Cortical Activities at the End of the Initial Reflexive Phase

In this study, the high-beta ERD was observed predominantly around the primary motor area. It peaked at 450 ms, which coincides roughly with N2 response. In general, high-beta ERDs have been identified for motor tasks other than the upright stance, in which they appear at the beginning of, or sometimes slightly prior to, the movement and sustain for the duration of the movement (Neuper and Pfurtscheller, 1996; Pfurtscheller et al., 1996; Pfurtscheller and Lopes da Silva, 1999; Jurkiewicz et al., 2006; Rossiter et al., 2014). Specifically, it was shown to appear continuously during sustained voluntary movements of hand and finger (Erbil and Ungan, 2007; Nakayashiki et al., 2014). A recent study showed that a smooth execution of a voluntary movement requires a decrease in the power of beta band (Heinrichs-Graham and Wilson, 2016), suggesting that beta ERDs are associated with the preparation of actions and related information processing to facilitate a motor execution. Smith et al. (2012) reported a pre-perturbation beta ERD at the Cz electrode that appears prior to the perturbation for predictable perturbations, and demonstrated that a large beta ERD in patients with Parkinson’s disease is associated with reduced adaptability to postural responses. However, the beta ERD identified in this study occurred clearly after the perturbation (after the major ERPs), and thus it may reflect a different functional role.

Related to the predictive response, one might wonder whether the EEG responses in the current study might also involve pre-perturbation components, due to the regularity of the sequence of perturbations (i.e., the perturbations were applied periodically every 20 s), by which the participants could predict and prepare for the onset of each perturbation. However, there was no significant difference between the baseline EEG during the pre-perturbation period and the EEG during quiet stance (Figure 6), implying no predictive cortical responses in the current study. However, it remains possible that the regularity of the sequence of perturbations used in this study may affect the “preparatory setting” of the spinal cord (Wälchli et al., 2017).

It has been clarified that beta ERDs appear even in passive (i.e., non-voluntary) motor tasks (Vinding et al., 2019) and also during the Go/NoGo tasks regardless of the motor selection, i.e., either Go or NoGo responses (Alegre et al., 2004; Wu et al., 2019). That is, beta ERD (and the subsequent ERS) is not necessarily accompanied with actual motor executions. It represents not only the information processing for afferent sensory signals, but also the decision-making process (Alegre et al., 2004; Jurkiewicz et al., 2006; Wu et al., 2019). The high-beta ERD in this study appeared when the CoP recovering toward the upright position caught and surpassed the CoM, after which CoP and CoM became close to each other. It also coincided with the period when the muscle activations became much smaller than those in the early phase of the postural response for *t* < 300 ms. This seemingly contradicts a typical appearance of beta ERD, because the high-beta ERD in this study was not accompanied with the major muscle activations for *t* < 300 ms. However, the major muscle activations with the large postural response for *t* < 300 ms could be considered as a stereotypical sequence of reflexes both at spinal and cortical levels, and the postural control after the early phase might require sophisticated information processing to achieve postural recovery, which might be represented in the high-beta ERD in this study. Similar to the P2 response, the high-beta ERD can be interpreted as a re-afferent phenomenon, as discussed in section “Neuroanatomical Interpretations of the Recovery Process” below with a neuroanatomical interpretation of the process. Moreover, the high-beta ERD after the early postural response could represent a preparation for possible actions after the perturbation, such as a stepping response in case that the perturbation is larger than a specific threshold. Specifically, this can be achieved by inhibiting the reflexive muscular reactions during the early phase of the postural response and by facilitating the cortico-spinal excitability (Solopova et al., 2003).

It is of interest to note that slightly before the high-beta ERD (*t* ∼ 270 ms), the alpha ERD appeared at the occipital region, which might correspond to the previously reported ERD at mu band (Jurkiewicz et al., 2006), and then expanded throughout the cortex until about *t* ∼ 1 s (Figure 3). Then, the theta ERD appeared specifically around Cz with the latency of *t* ∼ 800 ms and lasted for at least 3 s (Figures 3H, 5). During the time interval with the alpha ERD and theta ERD, the CoM was located close to or slightly ahead of the CoP, while the body was recovering toward the upright position. The theta ERD also exhibited a long-lasting property as in the high-beta ERS until the latency of *t* ∼ 3 s. The appearance of movement-related theta ERD is previously unreported to our best knowledge. Because it sustained for a long period in parallel with the beta rebound, it might play a coordinated role with the beta rebound in the sensory information processing.

### Long-Lasting High-Beta Rebound

A beta ERS, also known as a beta rebound, typically appears after completion of a movement, and often following the corresponding beta ERD (Neuper and Pfurtscheller, 1996; Pfurtscheller et al., 1996; Pfurtscheller and Lopes da Silva, 1999; Jurkiewicz et al., 2006). Consistently in this study, the high-beta ERS also appeared after the high-beta ERD. However, it began before the postural recovery was completed (Figures 3A–G,H), which is qualitatively different from previous studies for the motor tasks of upper extremities. The simplest interpretation of the difference is that the major phase of the postural response is completed within the early phase of the recovery process, and that the small residual response that remained during the beta ERS can be considered as *negligible*. If this is the case, we could consider that the high-beta ERS in this study is initiated after the movement as in the previous studies for the upper extremities, implying the same functional roles expressed by the high-beta ERS in this study and in the previous studies for upper extremities. That is, the cortical beta activity in humans represents a raise in the cortical activity levels that suppress voluntary movement (Gilbertson et al., 2005; Pastötter et al., 2008; van Wijk et al., 2009), while actively promoting postural maintenance (Gilbertson et al., 2005). The promotion of postural maintenance may be achieved through an up-regulation of relevant sensory inputs during and immediately after bursts of beta activity (Gilbertson et al., 2005; Androulidakis et al., 2006; Lalo et al., 2008).

An alternative and novel interpretation is required, if the small residual postural dynamics during the beta ERS are considered as *non-negligible*. If this is the case (and we though this is the case), it is inevitable to conclude that the beta ERS appeared before the perturbation-induced movement was completed, which is contradictory to the well-studied beta rebound after the movements. That is, the *non-negligible* residual error (the small deviation from the upright equilibrium) that was present during the beta ERS response would now play a role as a cause that drives the long-sustained high-beta ERS for about 3 s. In other words, if the residual error were neglected by the supraspinal mechanisms, it could not have driven the high-beta ERS for the long time, i.e., the high-beta ERS should have completed in a shorter duration than the 3 s. This logic would suggest that the small residual postural dynamics converging to the upright equilibrium are not simply the “almost equilibrated state,” but they should still be actively taken care of for achieving postural stabilization, which requires a certain amount of information processing of afferent sensory signals, leading to the generation of the long-lasting high-beta ERS.

We speculate that the long-lasting high-beta ERS at the late phase of postural recovery might be associated with active monitoring of the postural state in the framework of the intermittent control during human quiet standing (Bottaro et al., 2005, 2008; Asai et al., 2009). A major rationale behind this speculation is that the postural state point in the late phase of the recovery slowly approached the upright equilibrium along a linear-shaped trajectory in the 4th quadrant of position-velocity phase plane both in the human experiment (Figure 4) and in the numerical simulation of the intermittent control model (Figure 1). In the human experiment, the long-lasting high-beta ERS (as well as the theta ERD) appeared when the state point moved along this linear-shaped trajectory. In the intermittent control model, this linear-shaped trajectory is caused by the stable manifold of the model when the active feedback control is switched OFF in the OFF-region with a possibility of chattering between switch-ON and switch-OFF (section “Theoretical Background”). Despite that the postural state point has become close to the upright position in the late phase of the recovery, active monitoring of the postural state is necessary for the central nervous system to stabilize the upright posture, even if the active feedback control is not externalized, i.e., without chattering-like ON-OFF switching as in Figure 1A. As mentioned in section “Theoretical Background,” such active monitoring at the late phase of postural recovery might be accompanied by the sensorimotor information processing with a continuous attention by the central nervous system to determine whether the active control can remain switched OFF ongoingly, or the active control should be switched ON, either with or without the chattering-like ON-OFF switching. Because selecting (or switching between) OFF and ON requires a reliable estimate of the current state, active and continuous monitoring of the postural state plays an important role for achieving the intermittent postural control. Such active monitoring could be a cause of the high-beta ERS, as suggested by previous studies such that the beta ERS represents a processing of afferent sensory information (Jurkiewicz et al., 2006). Although this study is still preliminary and should be examined carefully in future, similarity between the ON-OFF switching in the intermittent model and the GO-NoGo selection in Go-NoGo tasks, and moreover, similarity in the beta ERD and the subsequent beta ERS in the postural recovery process (Figures 3H, 5) and in Go-NoGo tasks (Alegre et al., 2004; Zhang et al., 2008; Swann et al., 2009) would be enough initial evidence to speculate that the long-lasting beta ERS at the late phase of recovery might be associated with active monitoring of postural state along the stable manifold in conjunction with the intermittent control. See section “Neuroanatomical Interpretations of the Recovery Process” below for a neuroanatomical interpretation that might further support our speculation.

A recent study (Suzuki et al., 2020) advocated that the intermittency in the active feedback postural control used by healthy people for postural stabilization might be lost in patients with Parkinson’s disease, i.e., the active feedback control in the patients exhibited less ON-OFF switching. Perhaps, this is consistent with the attenuation in the beta ERS in patients with Parkinson’s disease due to impaired afferent sensory information processing (Vinding et al., 2019) and for decision-making motor tasks (Wu et al., 2019). This discussion should be considered as quite speculative due to limitations of this study, namely only nine health young participants and single perturbation parameters were considered, which hinders ERSP data from comparing with that for other conditions. Thus, further examinations on the long-lasting beta ERS, with elderly participants and patients with neurological diseases as well as with multiple perturbation conditions are necessary for elucidating the neural mechanisms of the long-lasting beta ERS. Ozdemir et al. (2018) showed age-dependent alterations in cortical activities, including a cortico-muscular coherence, at variety of frequency bands. Because differences in the degree of intermittency of EMGs of MG muscles between healthy people and patients with Parkinson’s disease are quite apparent (Yamamoto et al., 2011), it is highly expected that similar differences could be detected in cortical activities and cortico-spinal excitability (cortico-muscular coherence).

### Neuroanatomical Interpretations of the Recovery Process

Neural information processing to accomplish the postural recovery observed in this study is interpreted in this subsection, according to the neuroanatomical model of higher-order regulation of postural control (Takakusaki, 2017), which is proposed based on the knowledge mostly from animal (cat and monkey) experiments and some from human subjects, including neurological patients. We associate such a neurophysiological interpretation with the long-lasting high-beta ERS (beta rebound) at the late phase of the postural recovery, in conjunction with the intermittent control model. The following interpretation would become better understood by referring Figure 5 of Takakusaki (2017).

First, immediately after the perturbation-onset, the semicircular canal detects the perturbation-induced head acceleration, which evokes a stereotypical vestibular-spinal reflex (compensatory postural adjustment) that activates SO and then MG muscles with the shortest latency (Massion, 1992). The vestibular sensation is also transmitted to the cerebellum and the vestibular cortex (Shaikh et al., 2004), and then processed to activate the reticulospinal tract that induces co-contraction of the ankle muscles (including TA in addition to SO and MG) and other proximal muscles, making the trunk and lower extremities stiff for maintenance of vertical posture. The reflexive process up to this point might be associated with P1 and N1 of ERP, and the corresponding phase-locked ERS in the broad frequency band, other than the high-beta band.

In parallel with progress of the vestibular-spinal reflex, the vestibular sensation via S1, the visual sensation (head-movement-induced optical flow) and somatosensation of joints and muscles via S1 and the cerebellum are integrated at the posteroparietal cortex (near the Cz electrode) and the temporo-parietal cortex, in conjunction with the cerebellum, to establish a body schema of postural verticality (Barra et al., 2010; Lajoie et al., 2010; Pérennou et al., 2014). The body schema of postural verticality is then sent to the supplemental motor area (SMA)/premotor area (PM) for further processing by the cortico-basal ganglia and the cortico-cerebellar motor loops (Mori et al., 2003), perhaps to determine which muscle activations are facilitated and which are inhibited for stabilizing the posture (Jacobs et al., 2009).

The output of the motor loops from SMA/PM, as well as the inhibitory output of the motor loops from the basal ganglia, are transmitted along the descending pathway to the brainstem (pedunculopontine nucleus: PPN) to facilitate or inhibit specific sets of fibers of reticulospinal tract for flexor and extensor muscles (Takakusaki et al., 2003). Note that, for upper-extremity motor tasks, the major descending pathway is the pyramidal tract from the primary motor area, which processes the output of the motor loops from SMA/PM. The output from SMA/PM is also sent back to S1 and the posteroparietal cortex, as an efference copy of the descending motor command, to be compared with the reafference signal as the body schema of postural verticality represented in S1 and the posteroparietal cortex (Massion, 1992). Note that the upregulated output of the basal ganglia, as in patients with Parkinson’s disease, might result in the downregulation of hypotonia in PPN, leading to the co-contraction of antagonist muscles (Takakusaki et al., 2003). We speculate that the long-lasting high-beta ERS identified in this study, together with the theta ERD, are associated with the long-lasting neural expression of the body schema of postural verticality at S1 and the posteroparietal cortex and a process of matching between the body schema and reafference of the postural state, where they are required to be long-lasting due to slow mechanical dynamics of postural recovery, perhaps along the stable manifold of the unstable saddle-type upright equilibrium point. That is, those circuitries might fulfill a role of active monitoring (including matching between the efference copy and the afference information) ongoingly, either with (middle phase of the recovery after the vestibular-spinal reflex) or without (late phase of the recovery) EMG outputs, in order to be always ready for switching ON or OFF the intermittent feedback controller. We also speculate that the long-lasting nature of the high-beta ERS and the theta ERD might be associated with the long-lasting neuronal firing at the posteroparietal cortex, as shown in cats (Lajoie et al., 2010), which plays a role in the working memory for regulating ongoing motor behaviors.

### Limitation and Future Issues to Go Beyond a Speculation

As discussed above, this study should be considered as preliminary, because the experiment was conducted with only nine healthy young participants. Moreover, the interpretation associating the beta ERS in this study, the beta ERS in Go-NoGo paradigms and the active monitoring of postural dynamics during the OFF-period of active control is solely based on (1) similarity between a linear-shaped CoM trajectory on the phase plane in the experimental slow recovery of posture at the late phase and that in the intermittent control model along the stable manifold during the OFF-period of active control, (2) co-occurrence (coincidence) of the beta ERS and the slow recovery along the stable manifold (i.e., the OFF-period of active control), and (3) conceptual similarity between automatic ON/OFF selection in the intermittent postural control and voluntary Go/NoGo selection in Go-NoGo paradigms that also generate beta rebound (Alegre et al., 2004; Zhang et al., 2008; Swann et al., 2009). Although our unique attention to the important role played by the slow recovery along the stable manifold that appears during the OFF-period in the intermittent model (Bottaro et al., 2005, 2008; Asai et al., 2009; Nomura et al., 2020; Suzuki et al., 2020) motivated this study and has led to the identification of beta ERS, despite their long latency over 1.0 s and long duration over 3.0 s that have been hindering discovery of this response, the speculative interpretation of the beta ERS in this study should be examined carefully in the future studies.

One way of such examinations is to show a correlation between metrics that characterize the beta ERS and the OFF-period of the intermittent controller. To this end, we quantified latencies (timings) of the onset of the beta ERS and the onset of the OFF-period for eight out of nine individuals who exhibited the significantly large beta ERS, and tried to correlate between them. Because this is quite preliminary, we report a result only in the Supplementary Materials (Supplementary Figure 12), where we suggest a weak association between the onset of the beta ERS and the onset of the OFF-period (correlation coefficient of about 0.23). The small value of correlation might be primarily due to the small number of individuals participating in this study. Indeed, these two onset timings coincided quite well for six out of eight individuals (Supplementary Figure 12). Moreover, there is a known difficulty in characterizing the switching events between ON and OFF periods of the intermittent controller (Inoue and Sakaguchi, 2015; Nema et al., 2017), which might reduce the correlation. In this preliminary attempt (Supplementary Figures 12–20), we examined waveforms of the difference between CoM and CoP, i.e., ε(*t*) = CoM(*t*)−CoP(*t*) as a function of time *t*, and quantified the occurrence rate of the upward zero-crossing event for ε(*t*), because the upward zero-crossing events for ε(*t*) roughly represent the occurrences of switch-OFF events. More specifically, the CoM and CoP positions are largely separated with each other sometime after the perturbation, i.e., ε(*t*) is far from zero, and thus almost no zero-crossing event occurs. As the perturbed posture recovers to the quiet stance, the CoM and CoP positions become close to each other. Then, eventually, back and forth alternation in the relative position (in the anterior-posterior direction) with small amplitude appears intermittently during quiet stance, which is closely related to the so-called trembling component of postural sway (Zatsiorsky and Duarte, 2000) and the intermittent control (Bottaro et al., 2005, 2008). Time intervals during which ε(*t*) (and the trembling component) is slightly positive (CoM is slightly closer to the upright position than CoM) and stays at small values (CoM and CoP are close to each other) correspond to the OFF-periods of the intermittent controller. This is the reason why the upward zero-crossing events of ε(*t*) represent the switch-OFF events. The dominant frequency component and the upward zero-crossing rate during quiet stance are both about 0.9 Hz (Zatsiorsky and Duarte, 2000). In our speculation, we hypothesize that the active monitoring of postural state begins when the active controller is switched-OFF during postural recovery, which can be considered as a transition from the perturbed posture with reflexive control and almost no upward zero-crossing events of ε(*t*) to the quiet stance with the steady-state value of the upward zero-crossing rate of about 0.9 Hz. Because the upward zero-crossing rate of ε(*t*) was dropped from the steady-state value about 0.9 Hz to a small value sometime after the perturbation, and it recovered gradually toward the steady-state value, we defined the time of the onset of the OFF-period of the intermittent controller as the time when the upward zero-crossing rate of ε(*t*) reached its minimum value after the perturbation.

Although more rigorous exploration of correlations between ON/OFF switching of the intermittent controller and power of the beta ERS is important future issues for providing enough evidence that supports our speculation, it is not easy to modulate systematically the appearance of ON/OFF switching of the intermittent controller, because it is an automatic process, unlike the voluntarily Go/NoGo selections in Go-NoGo paradigms. Moreover, it is worthwhile to note that, when we examine correlations between ON/OFF switching of the intermittent controller and power of the beta ERS, we would not necessarily expect a direct co-modulation between ON/OFF switching and the beta ERS. This is because (1) the long-lasting beta ERS appears as an active monitoring process even without ON/OFF switching of the controller, but with the state point that simply moves along the stable manifold with keeping the active controller switched-OFF, and (2) ON/OFF switching in the intermittent control occurs at most 0.9 Hz and it would be far lower than it during the transition from the perturbed to quiet stance as mentioned above. Nevertheless, we believe that it would be possible to design experimental paradigms that can investigate the appearance of ON/OFF switching systematically, by which we probably obtain a clear correlation between the ON/OFF switching and the beta ERS. In our ongoing study, we are working on the EEG signals during quiet stance, without perturbation, in which the intermittent ON/OFF switching occurs stochastically along stochastic postural sway. In this case, we can detect both ON-actions and OFF-actions separately in response to a stochastic sequence of micro-falls (Loram et al., 2005) that happen during postural sway, and analyze the corresponding EEG responses separately. We expect the appearance of beta ERS for both ON and OFF actions, probably with a weaker power in the OFF actions compared to the ON actions, as in Go-NoGo paradigms with weaker beta ERS in NoGo responses compared to Go responses.

Until enough evidence for our speculative interpretation is provided, conventional interpretations for beta ERS during upper extremity motor tasks, including the one with “signaling the status-quo” by Engel and Fries (2010), would remain feasible also for the long-lasting beta ERS during upright stance.

## Data Availability Statement

The raw data supporting the conclusions of this article will be made available by the authors, without undue reservation.

## Ethics Statement

The studies involving human participants were reviewed and approved by the Ethical Committee of the Graduate School of Engineering Science at Osaka University. The patients/participants provided their written informed consent to participate in this study.

## Author Contributions

AN and TN conceived and designed the research and interpreted results of experiments. AN and YS performed the experiments. AN analyzed the data, prepared the figures, and drafted the manuscript. YS and MM advised on experimental and analytical methodologies. YS, MM, and TN edited and revised manuscript. All authors approved final version of manuscript.

## Conflict of Interest

The authors declare that the research was conducted in the absence of any commercial or financial relationships that could be construed as a potential conflict of interest.
