# Supplementary material for: Long-Lasting Event-Related Beta Synchronizations of Electroencephalographic Activity in Response to Support-Surface Perturbations During Upright Stance: A Pilot Study Associating Beta Rebound and Active Monitoring in the Intermittent Postural Control
Source: Front Syst Neurosci. 2021 May 21;15:660434. doi: 10.3389/fnsys.2021.660434 (PMC8175801; doi:10.3389/fnsys.2021.660434)
Supplement: Supplementary file 1 [file Data_Sheet_1.pdf]

Supplementary Material

S1. Definition of the triple-inverted pendulum model

We consider a triple-inverted pendulum model in the sagittal plane (Suzuki et al. 2012). See Fig. S1. The upper link of the model represents the head-arm-trunk (HAT link). The lower link represents the left and right lower extremities (LE link). The Foot link is fixed on the support-surface as in Fig. S1. Distal end of the LE link is connected to the Foot link by a pin joint, corresponding to the ankle joint. The distal end of HAT link and the proximal end of the LE link is connected by a pin joint, corresponding to the hip joint. Each link is considered as a rigid body with its mass distributed uniformly. The ankle joint angle θ_a and hip joint angle θ_h and body parameters are defined in Fig. S1.

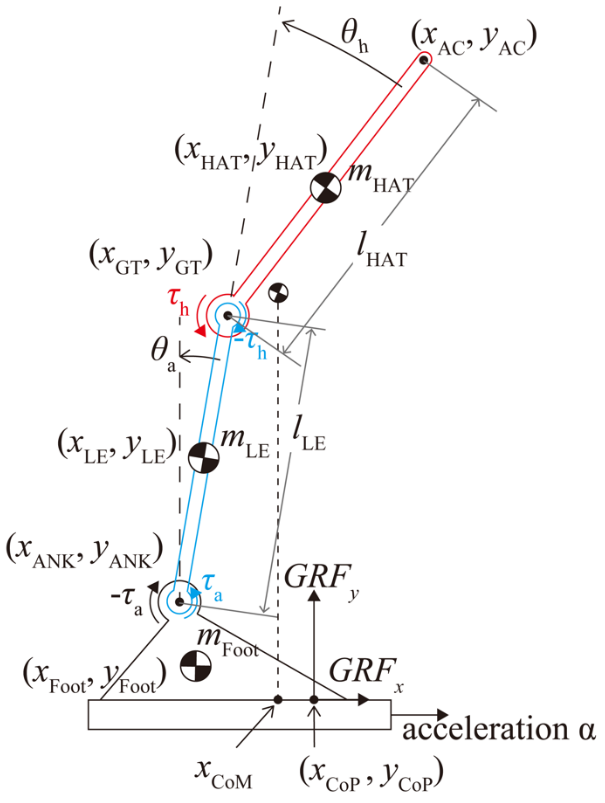

Figure S1. A triple-inverted pendulum with a Foot link fixed on the support-surface. The model consists of links of HAT, LE and Foot with a pin joint at the ankle and hip. See text in S1, S2 and S3.

S2. Joint angles estimation

The hip and ankle joint angles of each participant during standing were estimated using the inverted pendulum model described above and the marker positions attached to the ankles, the greater

trochanters and the acrominons of both of left and right sides of subject's body. Positions of those markers were measured by the motion-capture system in the global coordinate system, which were projected on the sagittal plane and averaged to obtain the position data for each of the ankle (x_{ANK}, y_{ANK}), greater trochanter (x_{GT}, y_{GT}) and acromion (x_{AC}, y_{AC}) on the sagittal plane. See Fig. S1. Then, θ_{a} and θ_{h} were calculated using the following equations

$$\theta_{\mathrm{a}} = -\tan^{-1}((x_{\mathrm{ANK}} - x_{\mathrm{GT}})/(y_{\mathrm{ANK}} - y_{\mathrm{GT}})),$$

$$\theta_{\mathrm{h}} = -\tan^{-1}((x_{\mathrm{GT}} - x_{\mathrm{AC}})/(y_{\mathrm{GT}} - y_{\mathrm{AC}})) - \theta_{\mathrm{a}},$$

in which the counterclockwise rotation (plantar flexion for the ankle joint and extension for the hip joint) was defined as the positive direction.

S3. CoM estimation

Horizontal position of the total body center of mass (CoM) in the AP direction during standing was estimated as

$$x_{\mathrm{CoM}} = \frac{m_{\mathrm{LE}} x_{\mathrm{LE}} + m_{\mathrm{HAT}} x_{\mathrm{HAT}}}{m_{\mathrm{LE}} + m_{\mathrm{HAT}}},$$

$$x_{\mathrm{LE}} = \left(\frac{l_{\mathrm{LE}}}{2}\right) \sin\theta_{\mathrm{a}},$$

$$x_{\mathrm{HAT}} = l_{\mathrm{LE}} \sin\theta_{\mathrm{a}} + \left(\frac{l_{\mathrm{HAT}}}{2}\right) \sin(\theta_{\mathrm{a}} + \theta_{\mathrm{h}}),$$

where l_{HAT} and l_{LE} represent the lengths of HAT and LE links, respectively as in Section 2 of the main text. l_{HAT} and l_{LE} were determined, respectively, by the mean distance between time series of two positions (x_{GT}, y_{GT}) and (x_{AC}, y_{AC}) and by that between (x_{ANK}, y_{ANK}) and (x_{GT}, y_{GT}), for each participant. We assumed that the CoM of each link is located at the middle point of the link. See Fig. S1.

S4. CoP positions relative to the foot

In the perturbed condition, the foot position shifted backward in response to every perturbation by the amount of the backward shift of the belt. Because the force plate and its local coordinate system were fixed in the global coordinate system independent of the moving belt, a time-profile of the recorded CoP position during the 7 min, denoted by $x_{\mathrm{CoP}}(t)$ in the global coordinate system as a function of time t, was the sum of a time-profile of the actual postural response $x_{\mathrm{CoP}}^{\mathrm{L}}(t)$ in the local coordinate system fixed on the belt (the CoP position relative to the foot) and a series of step-wise backward shifts of the belt that was measured as the position of the ankle joint $x_{\mathrm{ANK}}(t)$ both in the global coordinate system, i.e., $x_{\mathrm{CoP}}(t) = x_{\mathrm{CoP}}^{\mathrm{L}}(t) + x_{\mathrm{ANK}}(t)$. Thus, we could obtain the time series of CoP positions relative to the foot as $x_{\mathrm{CoP}}^{\mathrm{L}}(t) = x_{\mathrm{CoP}}(t) - x_{\mathrm{ANK}}(t)$, which represents the actual postural response to the perturbation.

S5. Equations of motion of the inverted pendulum model for inverse dynamics

The equation of motions in the global coordinate system for each of the three links, namely, the HAT link, the LE link and the Foot link of the triple-inverted pendulum model, are as follows (Suzuki et al. 2012). See Fig. S1.

The HAT link:

$$m_{\mathrm{HAT}} \frac{\mathrm{d}^2}{\mathrm{dt}^2} x_{\mathrm{HAT}} = F_x$$

$$m_{\mathrm{HAT}} \frac{\mathrm{d}^2}{\mathrm{dt}^2} y_{\mathrm{HAT}} = F_y - m_{\mathrm{HAT}} g$$

$$I_{\mathrm{HAT}} \frac{\mathrm{d}^2}{\mathrm{dt}^2} (\theta_{\mathrm{a}} + \theta_{\mathrm{h}}) = (x_{\mathrm{GT}} - x_{\mathrm{HAT}}) F_y - (y_{\mathrm{GT}} - y_{\mathrm{HAT}}) F_x + \tau_{\mathrm{h}}$$

The LE link:

$$m_{\mathrm{LE}} \frac{\mathrm{d}^2}{\mathrm{dt}^2} x_{\mathrm{LE}} = R_x - F_x$$

$$m_{\mathrm{LE}} \frac{\mathrm{d}^2}{\mathrm{dt}^2} y_{\mathrm{LE}} = R_y - F_y - m_{\mathrm{LE}} g$$

$$I_{\mathrm{LE}} \frac{\mathrm{d}^2}{\mathrm{dt}^2} \theta_{\mathrm{a}} = (x_{\mathrm{GT}} - x_{\mathrm{LE}})(-F_y) - (y_{\mathrm{GT}} - y_{\mathrm{LE}})(-F_x) + (x_{\mathrm{ANK}} - x_{\mathrm{LE}}) R_y - (y_{\mathrm{ANK}} - y_{\mathrm{LE}}) R_x + \tau_{\mathrm{a}} - \tau_{\mathrm{h}}$$

The Foot link:

$$m_{\mathrm{Foot}} \frac{\mathrm{d}^2}{\mathrm{dt}^2} x_{\mathrm{Foot}} = GRF_x - R_x$$

$$m_{\mathrm{Foot}} \frac{\mathrm{d}^2}{\mathrm{dt}^2} y_{\mathrm{Foot}} = GRF_y - R_y - m_{\mathrm{Foot}} g$$

$$I_{\mathrm{Foot}} \frac{\mathrm{d}^2}{\mathrm{dt}^2} \theta_{\mathrm{f}} = (x_{\mathrm{ANK}} - x_{\mathrm{Foot}})(-R_y) - (y_{\mathrm{ANK}} - y_{\mathrm{Foot}})(-R_x) + (x_{\mathrm{CoP}} - x_{\mathrm{Foot}}) GRF_y - (y_{\mathrm{CoP}} - y_{\mathrm{Foot}}) GRF_x - \tau_{\mathrm{a}}$$

I_{HAT}, I_{LE} and I_{Foot} are the inertia moments of the HAT link, the LE link, and the Foot link, respectively. F_x and F_y are the forces applied at the hip joint from the LE link to the HAT link. R_x and R_y are the forces applied at the ankle joint from the Foot link to the LE link. m_{HAT}, m_{LE} and m_{Foot} are the masses of the HAT link, the LE link and the Foot link, respectively. They were estimated from the total body weight of each participant using the following statistical formula. $m_{\mathrm{HAT}}: m_{\mathrm{LE}}: m_{\mathrm{Foot}} = 0.62: 0.35: 0.03$ (Suzuki et al. 2012).

We performed the inverse dynamics analysis by eliminating unknown variables (F_x, F_y, R_x, R_y) from the equations of motion. Equation of motion for the Foot link was used for estimating the ankle joint torque, because it is more accurate to estimate the joint torque using the ground reaction force. Then, joint torques exerted on the ankle joint τ_{a} and the hip joint τ_{h} were estimated as follows.

$$\tau_{\mathrm{a}} = (x_{\mathrm{CoP}} - x_{\mathrm{ANK}})GRF_y - (y_{\mathrm{CoP}} - y_{\mathrm{ANK}})GRF_x + (x_{\mathrm{Foot}} - x_{\mathrm{ANK}})(-m_{\mathrm{Foot}}g) - (y_{\mathrm{Foot}} - y_{\mathrm{ANK}})(-m_{\mathrm{Foot}}\alpha)$$

$$\tau_{\mathrm{h}} = \frac{1}{6} m_{\mathrm{LE}} l_{\mathrm{LE}}^2 \ddot{\theta}_{\mathrm{a}} - l_{\mathrm{LE}} \left(\frac{m_{\mathrm{LE}}}{2} + m_{\mathrm{Foot}} \right) (g \sin\theta_{\mathrm{a}} + \alpha\cos\theta_{\mathrm{a}}) + l_{\mathrm{LE}} \left(GRF_y \sin\theta_{\mathrm{a}} + GRF_x \cos\theta_{\mathrm{a}} \right) + \tau_{\mathrm{a}}$$

where α represents the acceleration of the support-surface (perturbation), which was -4 m/s^2 for the first half 100 ms and 4 m/s^2 for the latter half 100 ms. We assumed that the Foot link is fixed on the moving support-surface with no translational and rotational movement ($\ddot{x}_{\mathrm{Foot}} = \alpha$, $\ddot{y}_{\mathrm{Foot}} = 0$, $\ddot{\theta}_{\mathrm{f}} = 0$). Moreover, we assumed $(x_{\mathrm{Foot}} - x_{\mathrm{ANK}}) = 0.02$ m, $(y_{\mathrm{Foot}} - y_{\mathrm{ANK}}) = -y_{\mathrm{ANK}}/2$ to compute τ_{a}.

S6. Intermittent posture control model for numerical simulations

The equation of motion used for the numerical simulation in Section 2 of the main text is described here. Since the degree of freedom of the triple inverted pendulum with the spatially fixed Foot link is 2 (i.e., the ankle joint angle θ_{a} and the hip joint angle θ_{h}), the non-redundant equations of motion for θ_{a} and θ_{h} can be derived from the equations of motion of HAT link and LE link of described above in S5 (Suzuki et al. 2012; Morasso et al., 2019) as follows.

$$\boldsymbol{M}(\boldsymbol{\theta})\ddot{\boldsymbol{\theta}} + \boldsymbol{C}(\boldsymbol{\theta}, \dot{\boldsymbol{\theta}}) + \boldsymbol{G}(\boldsymbol{\theta}) = \boldsymbol{T}$$

where $\boldsymbol{\theta} = (\theta_{\mathrm{a}}, \theta_{\mathrm{h}})^T$, $\boldsymbol{T} = (\tau_{\mathrm{a}}, \tau_{\mathrm{h}})^T$, and $\boldsymbol{M}(\boldsymbol{\theta})$, $\boldsymbol{C}(\boldsymbol{\theta}, \dot{\boldsymbol{\theta}})$ and $\boldsymbol{G}(\boldsymbol{\theta})$ are the inertia matrix, the term of centrifugal and Coriolis forces and the gravitational toppling torque as in described in Section 2 of the main text. These terms are mathematically expressed as

$$\boldsymbol{M}(\boldsymbol{\theta}) = \begin{pmatrix} m_{11}(\boldsymbol{\theta}) & m_{12}(\boldsymbol{\theta}) \\ m_{21}(\boldsymbol{\theta}) & m_{22}(\boldsymbol{\theta}) \end{pmatrix},$$
$$m_{11}(\boldsymbol{\theta}) = (I_{\mathrm{LE}} + m_{\mathrm{LE}} r_{\mathrm{LE}}^2) + m_{\mathrm{HAT}} l_{\mathrm{LE}}^2 + (I_{\mathrm{HAT}} + m_{\mathrm{HAT}} r_{\mathrm{HAT}}^2) + 2 m_{\mathrm{HAT}} l_{\mathrm{LE}} r_{\mathrm{HAT}} \cos\theta_{\mathrm{h}},$$
$$m_{12}(\boldsymbol{\theta}) = (I_{\mathrm{HAT}} + m_{\mathrm{HAT}} r_{\mathrm{HAT}}^2) + m_{\mathrm{HAT}} l_{\mathrm{LE}} r_{\mathrm{HAT}} \cos\theta_{\mathrm{h}},$$
$$m_{12}(\boldsymbol{\theta}) = (I_{\mathrm{HAT}} + m_{\mathrm{HAT}} r_{\mathrm{HAT}}^2) + m_{\mathrm{HAT}} l_{\mathrm{LE}} r_{\mathrm{HAT}} \cos\theta_{\mathrm{h}},$$
$$m_{22}(\boldsymbol{\theta}) = I_{\mathrm{HAT}} + m_{\mathrm{HAT}} r_{\mathrm{HAT}}^2,$$
$$\boldsymbol{C}(\boldsymbol{\theta}, \dot{\boldsymbol{\theta}}) = \begin{pmatrix} -m_{\mathrm{HAT}} l_{\mathrm{LE}} r_{\mathrm{HAT}} \left(2\dot{\theta}_{\mathrm{a}}\dot{\theta}_{\mathrm{h}} + \dot{\theta}_{\mathrm{h}}^2 \right) \sin\theta_{\mathrm{h}} \\ m_{\mathrm{HAT}} l_{\mathrm{LE}} r_{\mathrm{HAT}} \dot{\theta}_{\mathrm{a}}^2 \sin\theta_{\mathrm{h}} \end{pmatrix},$$
$$\boldsymbol{G}(\boldsymbol{\theta}) = \begin{pmatrix} -(m_{\mathrm{HAT}} l_{\mathrm{LE}} + m_{\mathrm{LE}} r_{\mathrm{LE}}) g \sin\theta_{\mathrm{a}} - m_{\mathrm{HAT}} r_{\mathrm{HAT}} g \sin(\theta_{\mathrm{a}} + \theta_{\mathrm{h}}) \\ -m_{\mathrm{HAT}} r_{\mathrm{HAT}} g \sin(\theta_{\mathrm{a}} + \theta_{\mathrm{h}}) \end{pmatrix}.$$

The ankle torque $\boldsymbol{T}$ can be decomposed into the following four types.

$$\boldsymbol{T} = \boldsymbol{T}^{\mathbf{pass}} + \boldsymbol{T}^{\mathbf{act}} + \boldsymbol{T}^{\mathbf{reflex}} + \boldsymbol{T}^{\mathbf{pert}} + \boldsymbol{T}^{\mathbf{n}}$$

$\boldsymbol{T}^{\mathbf{pert}}$ is the joint torque generated by the acceleration α of the support-surface, and it is expressed as follows.

$$\boldsymbol{T}^{\mathrm{pert}}(\boldsymbol{\theta}) = \begin{pmatrix} (m_{\mathrm{HAT}} r_{\mathrm{HAT}} \cos(\theta_{\mathrm{a}} + \theta_{\mathrm{h}}) + (m_{\mathrm{HAT}} l_{\mathrm{LE}} + m_{\mathrm{LE}} r_{\mathrm{LE}}) \cos\theta_{\mathrm{a}})\alpha \\ m_{\mathrm{HAT}} r_{\mathrm{HAT}} \cos(\theta_{\mathrm{a}} + \theta_{\mathrm{h}})\alpha \end{pmatrix}.$$

$\boldsymbol{T}^{\mathbf{pass}}$, $\boldsymbol{T}^{\mathbf{act}}$, $\boldsymbol{T}^{\mathbf{reflex}}$, and $\boldsymbol{T}^{\mathbf{n}}$ defined in Section 2 of the main text are represented as follows.

$\boldsymbol{T}^{\mathbf{pass}} = (\tau_{\mathrm{a}}^{\mathrm{pass}}, \tau_{\mathrm{h}}^{\mathrm{pass}})^T$ represents the passive joint torque, which is determined by the torsional elasticity and viscosity of the ankle joint (K_{a} and B_{a}) and those of the hip joint (K_{h} and B_{h}) for a given set of constant muscle tonuses that are determined in a feedforward manner for quiet stance. That is, $\boldsymbol{T}^{\mathbf{pass}}$ is simply modeled by linear springs and dampers as follows.

$$\boldsymbol{T}^{\mathbf{pass}} = \begin{pmatrix} \tau_{\mathrm{a}}^{\mathrm{pass}} \\ \tau_{\mathrm{h}}^{\mathrm{pass}} \end{pmatrix} = -\begin{pmatrix} K_{\mathrm{a}}\theta_{\mathrm{a}} + B_{\mathrm{a}}\dot{\theta}_{\mathrm{a}} \\ K_{\mathrm{h}}\theta_{\mathrm{h}} + B_{\mathrm{h}}\dot{\theta}_{\mathrm{h}} \end{pmatrix}$$

$\boldsymbol{T}^{\mathbf{act}} = (\tau_{\mathrm{a}}^{\mathrm{act}}, \tau_{\mathrm{h}}^{\mathrm{act}})^T$ represents the active feedback control torque for stabilizing quiet stance. We assume that $\boldsymbol{T}^{\mathbf{act}}(t)$ at time t is determined by the supraspinal circuitry that processes time-delayed sensory feedback information on the posture $\boldsymbol{\theta}(t-\Delta)$ and $\dot{\boldsymbol{\theta}}(t-\Delta)$, where Δ is a feedback time-delay for quiet stance (Δ=200 ms). For simplicity, we assume that $\boldsymbol{T}^{\mathbf{act}}$ is operated only at the ankle joint as $\tau_{\mathrm{a}}^{\mathrm{act}}$.

$$\boldsymbol{T}^{\mathbf{act}} = \begin{pmatrix} \tau_{\mathrm{a}}^{\mathrm{act}} \\ 0 \end{pmatrix},$$

$$\tau_{\mathrm{a}}^{\mathrm{act}}(t) = \begin{cases} 0 & \text{if } (\theta_{\mathrm{CoM}}(t-\Delta), \dot{\theta}_{\mathrm{CoM}}(t-\Delta))^T \in \mathcal{D}_{\mathrm{OFF}} \\ P\theta_{\mathrm{CoM}}(t-\Delta) + D\dot{\theta}_{\mathrm{CoM}}(t-\Delta) & \text{if } (\theta_{\mathrm{CoM}}(t-\Delta), \dot{\theta}_{\mathrm{CoM}}(t-\Delta))^T \in \mathcal{D}_{\mathrm{ON}} \end{cases}$$

That is, $\tau_{\mathrm{a}}^{\mathrm{act}}$ is switched OFF, if the delay affected state point is located in the OFF-region denoted by $\mathcal{D}_{\mathrm{OFF}}$ (see Fig. 1 and Fig. 2 in the main text). It is switched ON, and operates according to the proportional (P) and derivative (D) feedback controller (delayed PD feedback controller), if the delay affected state point is located in the ON-region denoted by $\mathcal{D}_{\mathrm{ON}}$.

$\boldsymbol{T}^{\mathbf{reflex}}$ represents a reflexive feedback control torque, which is operated in response to the perturbation only for a short duration at the early phase of postural recovery. We assume for simplicity that the reflexive control also operates only at the ankle joint, which is defined as

$$\boldsymbol{T}^{\mathbf{reflex}} = \begin{pmatrix} \tau_{\mathrm{a}}^{\mathrm{reflex}} \\ 0 \end{pmatrix},$$

$$\tau_{\mathrm{a}}^{\mathrm{reflex}}(t) = P^{\mathrm{reflex}}\theta_{\mathrm{CoM}}(t-\Delta_{\mathrm{reflex}}) + D^{\mathrm{reflex}}\dot{\theta}_{\mathrm{CoM}}(t-\Delta_{\mathrm{reflex}}),$$

where Δ_{reflex}=50 ms is another feedback time-delay. Δ_{reflex} is shorter than Δ, in consideration of stereotypical nature of the spinal and supraspinal reflex arcs. We assume that the reflexive control operates transiently only for a short period of time *(*onset at *t*=Δ_{reflex}=50 ms, and offset at *t*=250 ms in Fig. 1A or *t*=230 ms in Fig. 1B*)* in response to the perturbation. Moreover, we assume large gains of the reflexive controller *(*$P^{\mathrm{reflex}} = 327$ Nm/rad and $D^{\mathrm{reflex}} = 50$ Nms/rad*)*. Note that the intermittent PD feedback control $\tau_{\mathrm{a}}^{\mathrm{act}}$ is not operated when the reflexive control is in action.

$\boldsymbol{T}^{\mathbf{n}}$ represents the torque noise. Gaussian white noise was used as $\boldsymbol{T}^{\mathbf{n}}$ when we considered a motor noise, and it was equal to zero when no noise was considered.

S7. EEG signal preprocessing

Preprocessing, denoising and analysis of EEG signals were conducted using EEGLAB (Delorme and Makeig 2004; Loo et al. 2019). First, EEG data were down-sampled to 1,000 Hz to reduce the

computational time required for further processing. A zero-lag high-pass first-order Butterworth filter with a cutoff frequency of 1 Hz was applied to optimize signal-to-noise ratio (Winkler et al. 2015). We then removed data from noisy electrodes because we used the average reference potential method (Bigdely-Shamlo et al. 2015), for which a large noise in one electrode would affect the other electrodes. In this study, an electrode was considered as largely noisy if the correlation coefficients between the surrounding electrodes were smaller than 0.8. The average number of electrodes rejected in single trials was 0.28 out of 32.

We performed the artifact subspace reconstruction (ASR), which is a method for denoising EEG signals using principal component analysis (PCA) (Mullen et al. 2015). Steps of the ASR processing are summarized as follows: (1) Find and extract less noisy time intervals from the 32-dimenional EEG time-series and apply PCA to the extracted data to define the template dataset with the basis vectors of the principal components (i.e., principal axes) for expressing other EEG signals in general. (2) Decompose all EEG time-series data within a time-window of 1.0 s into the principal axes to obtain the magnitude of variance for each principal axis. (3) Remove components if their variances were 20 times larger compared to those of the corresponding components for the template data, and reconstruct the EEG for this window using other remaining components. (4) Repeat the decomposition while shifting the time-window with 66% overlap (Mullen et al. 2015). After ASR, data for the removed electrodes were spatially and linearly interpolated using the data from the surrounding electrodes (Bigdely-Shamlo et al. 2015). Finally, re-referencing was performed based on the averaged potential of all electrodes, which eliminates a tendency for electrodes around CPz as the reference electrode to exhibit smaller amplitudes compared with the other electrodes.

To remove muscle-activity and eye-blinking related artifacts from the EEG data, the independent component analysis (ICA) was performed. Artifact components were defined using the method described by Bruijn et al. (2015). That is, any independent component (IC) was considered to be affected by EMG activities of muscles, if its average power in the frequency band between 50 to 100 Hz was larger compared to that of the alpha band (8 to 12 Hz) and/or the low-beta band (13 to 20 Hz). Moreover, any IC was considered to be affected by eye-blinking originated electrooculograms (EOGs), if its central frequency was under 3 Hz and the corresponding topographic-map-representation was distributed around the forehead. The mean number of removed EMG and EOG components for single trials was 2.3 and 1.6 out of 32, respectively. After removing those artifact ICs, the remaining ICs were re-mapped onto the electrodes. The re-mapped EEG data in the time interval from 5 s before the perturbation until 15 s after the perturbation was then used as an epoch.

To confirm that no apparent contamination of EMG signals due to activity of the craniofacial muscles into cleaned EEG, power spectra of all electrodes were examined, particularly for 0 to 500 ms time interval after the perturbation, and confirmed monotonic decreasing shape, which is typical for EMG-noise-free EEG signals, in all spectra (Fig. S2).

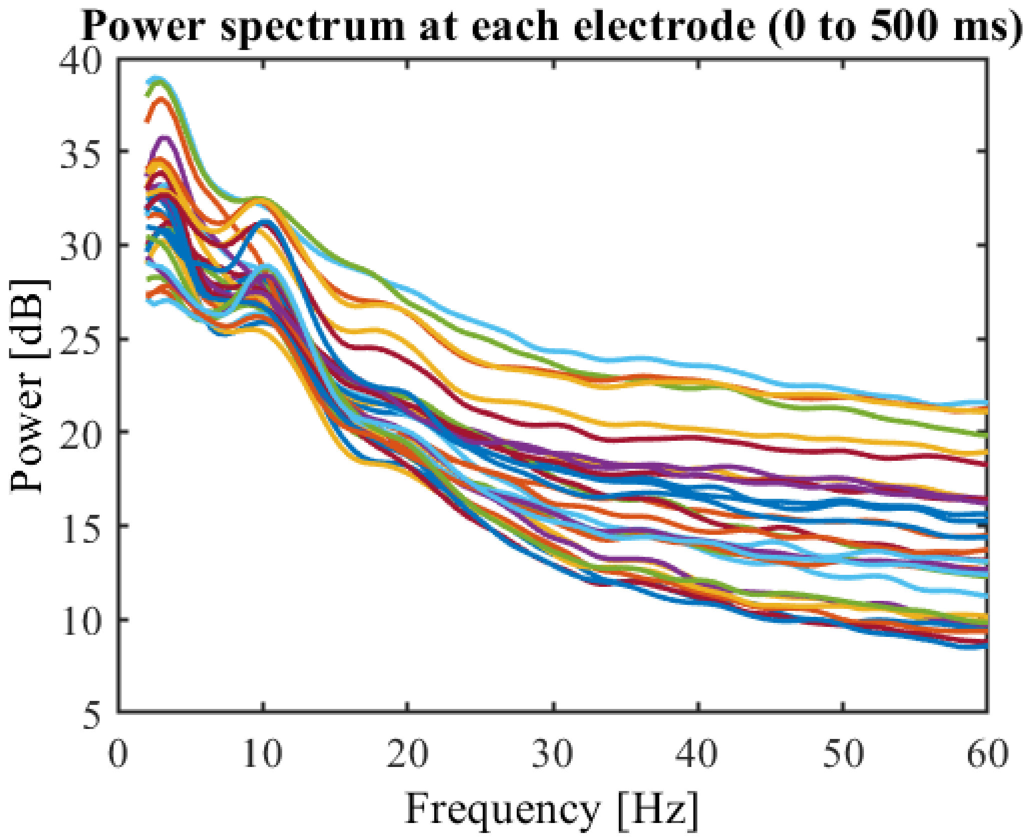

Figure S2. EEG power spectra for each of 31 electrodes, estimated for data between 0 and 500 ms after perturbation

S8. Wavelet transformation for computing ERSPs

Outcomes of time-frequency analysis for each epoch for each electrode were summarized by averaging them over all epochs, for each participant as well as across participants to obtain ERSPs (Makeig 1993). For each epoch, we performed a time-frequency analysis using the wavelet transform with the Morlet wavelet ψ(t) defined as

$$\psi(t) = \frac{1}{\sqrt{2\pi\sigma^2}} e^{-\frac{t^2}{2\sigma^2}} e^{j2\pi t},$$

where the number of cycles for the continuous wavelet transformation that defines σ was increased gradually from 3 to 24 corresponding to the range of 3 to 60 Hz, which covers all relevant EEG frequency bands, i.e., theta (4-7 Hz), alpha (8-12 Hz), low-beta (13-20 Hz), high-beta (21-30 Hz) and gamma (40-60 Hz).

S9. Responses of the perturbation in each subject

We confirmed that there was no outlier of ERSP by plotting an ERSP map for each trial and for averaged ERSP across trails within each subject (Figs. S3-S11). We also confirmed that beta rebound appeared within the frequency band of 21 - 30 Hz (high-beta) in all participants, except subject 9 for

whom almost no beta rebound was identified. As can be confirmed in Figs. S3-S11, the beta rebound (with statistically significant larger power than the baseline) sustained for a long period of time in ERSP of each participant. That is, the long-lasting property was found not only in the grand averaged ERSP over the participant as shown in the main text (Fig. 3), but also in ERSP for each participant.

S10. Association between beta ERS and intermittent control

To show a correlation between metrics that characterize the beta ERS and the OFF-period of the intermittent controller, we quantified latencies (timings) of the onset of the beta ERS and the onset of the OFF-period for eight out of nine individuals who exhibited the significantly large beta ERS, and tried to correlate between them.

To this end, we examined waveforms of the difference between CoM and CoP, i.e., $\varepsilon(t) = \mathrm{CoM}(t) - \mathrm{CoP}(t)$ as a function of time t, and quantified the occurrence rate of the upward zero-crossing event for $\varepsilon(t)$, because the upward zero-crossing events for $\varepsilon(t)$ roughly represent the occurrences of switch-OFF events. Specifically, occurrence rate of the upward zero-crossing event for $\varepsilon(t)$ was calculated by the following procedure. Epochs of CoM and CoP time series were extracted in the range of -4 s to 9 s. To reduce effects of high-frequency noise in the optical motion-capture-markers, a 4th-order Butterworth filter with a cutoff frequency of 5 Hz was applied to the CoM time series. After that, $\varepsilon(t) = \mathrm{CoM}(t) - \mathrm{CoP}(t)$ was calculated (Figs. S13-S20 upper panel). For detecting upward zero-crossing events of $\varepsilon(t)$, the upward zero-crossing events that occurred within intervals less than 50 ms were neglected, because they represent a situation such that CoM and CoP are in tangential, rather than intersectional. After the detection of all upward zero-crossing events for each epoch, a sequence of the instantaneous frequencies (inverse of inter-event intervals) was calculated. Finally, we obtained 40 time series of the instantaneous frequency for each subject. Subject 9 was excluded from this analysis, because beta ERS of this subject was weak and not significantly prominent. The intersection rate was calculated by averaging 40 instantaneous frequency time series and smoothing them using an unweighted zero-lag moving average with a window width of 1s. In this way, we obtained a smooth curve of the intersection rate, which represents the switched-OFF rate as a function of time for each subject. We defined the time of the onset of the OFF-period of the intermittent controller as the time when the smooth curve of the intersection rate reached its minimum value after the perturbation. See the main text.

The onset of beta ERS was detected as follows. First, the averaged power time series of high-beta ERS was obtained by averaging powers within 21 to 30 Hz at each time instant. The value of 90 percentile of the averaged power time series for each subject was set as the threshold value for that subject, and the time when the threshold value was exceeded was defined as the onset of the beta ERS. More specifically, the onset time of the long-lasting beta ERS was detected from the averaged power time series 0.8 s after the perturbation onset.

Then, the onset time of switch-OFF of the active controller and the onset time of the beta ERS was plotted for eight participants as a serial correlation diagram (Fig. S12), and the correlation between them was quantified by the correlation coefficient.

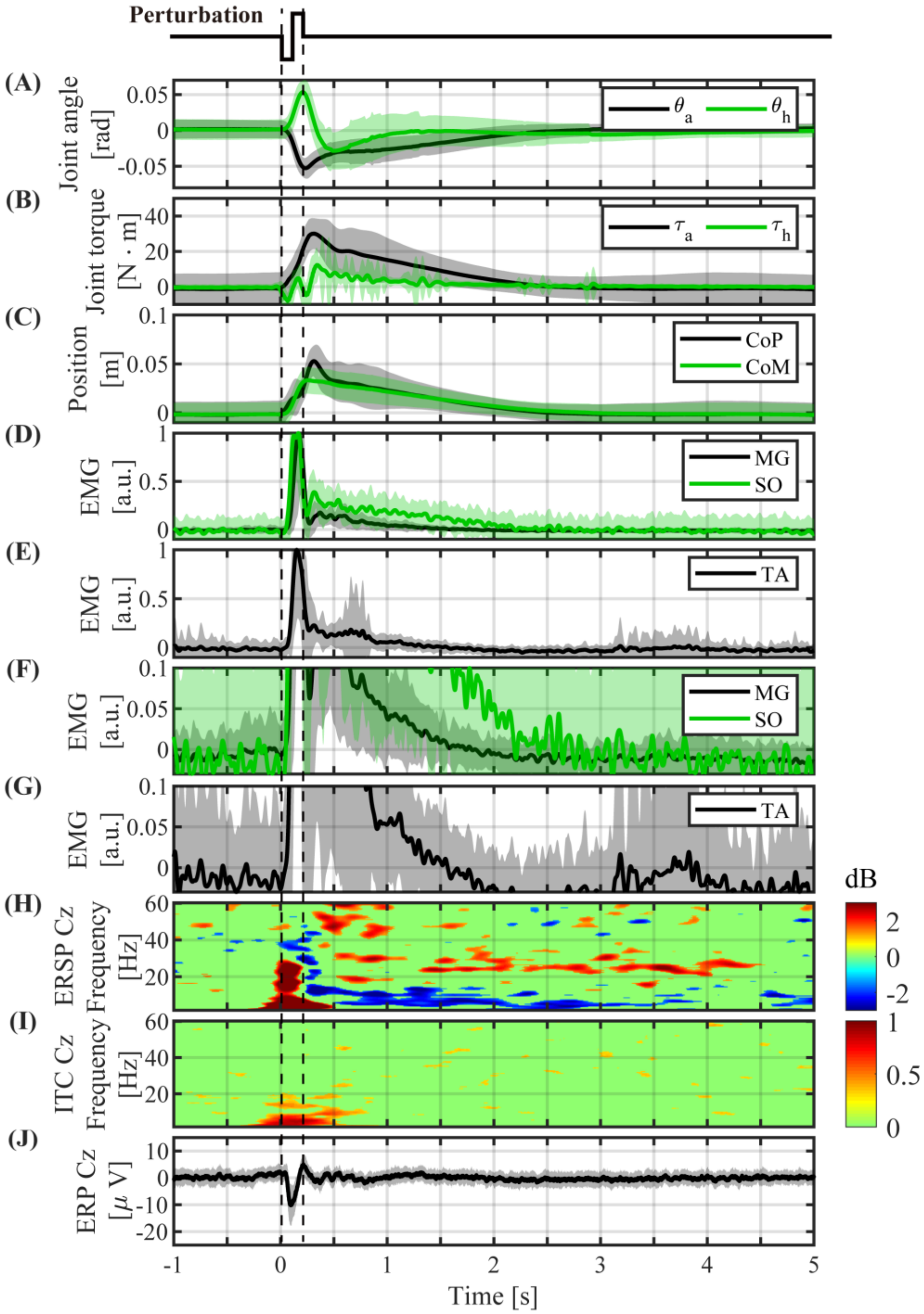

Figure S3. Event-locked average profiles of Subject 1 triggered by the perturbation-onset. (A) ankle and hip joint angles, (B) joint torques, (C) CoP and CoM positions, (D) normalized EMGs of Medial-Gastrocnemius and Soleus, (E) normalized EMG of Tibialis Anterior, (F) magnification of (D), (G) magnification of (E), (H) ERSP of Cz electrode, (I), ITC of Cz electrode, (J) ERP of Cz electrode. The light color shaded area in each of (A)-(G) and (J) is the standard deviation, representing the distribution across all perturbed trials of Subject 1. Nonsignificant differences from baseline (bootstrap statistics, $p > 0.05$) were set to 0 dB (green) in (H) and 0 (green) in (I).

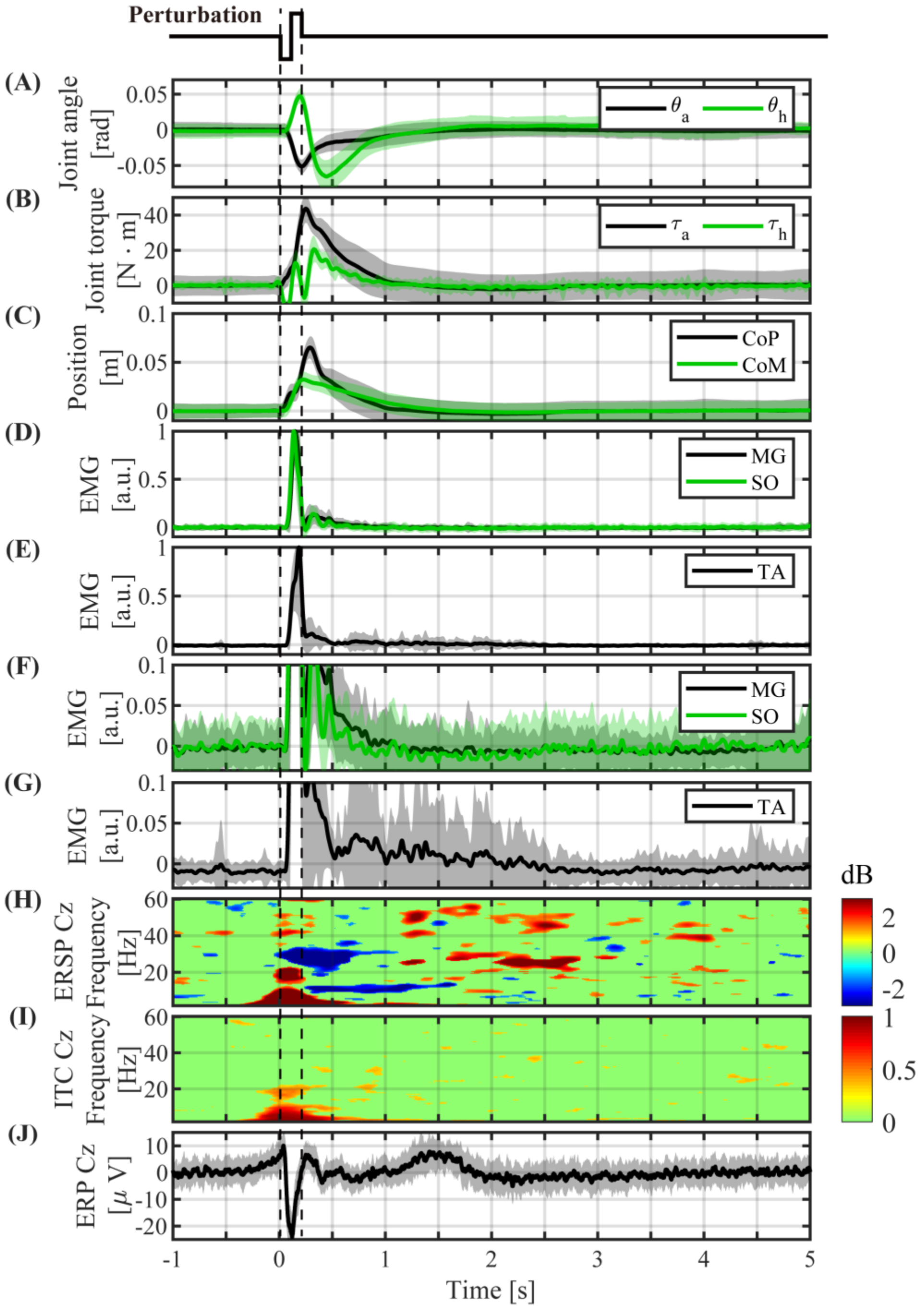

Figure S4. Event-locked average profiles of Subject 2 triggered by the perturbation-onset. (A) ankle and hip joint angles, (B) joint torques, (C) CoP and CoM positions, (D) normalized EMGs of Medial-Gastrocnemius and Soleus, (E) normalized EMG of Tibialis Anterior, (F) magnification of (D), (G) magnification of (E), (H) ERSP of Cz electrode, (I), ITC of Cz electrode, (J) ERP of Cz electrode. The light color shaded area in each of (A)-(G) and (J) is the standard deviation, representing the distribution across all perturbed trials of Subject 2. Nonsignificant differences from baseline (bootstrap statistics, $p > 0.05$) were set to 0 dB (green) in (H) and 0 (green) in (I).

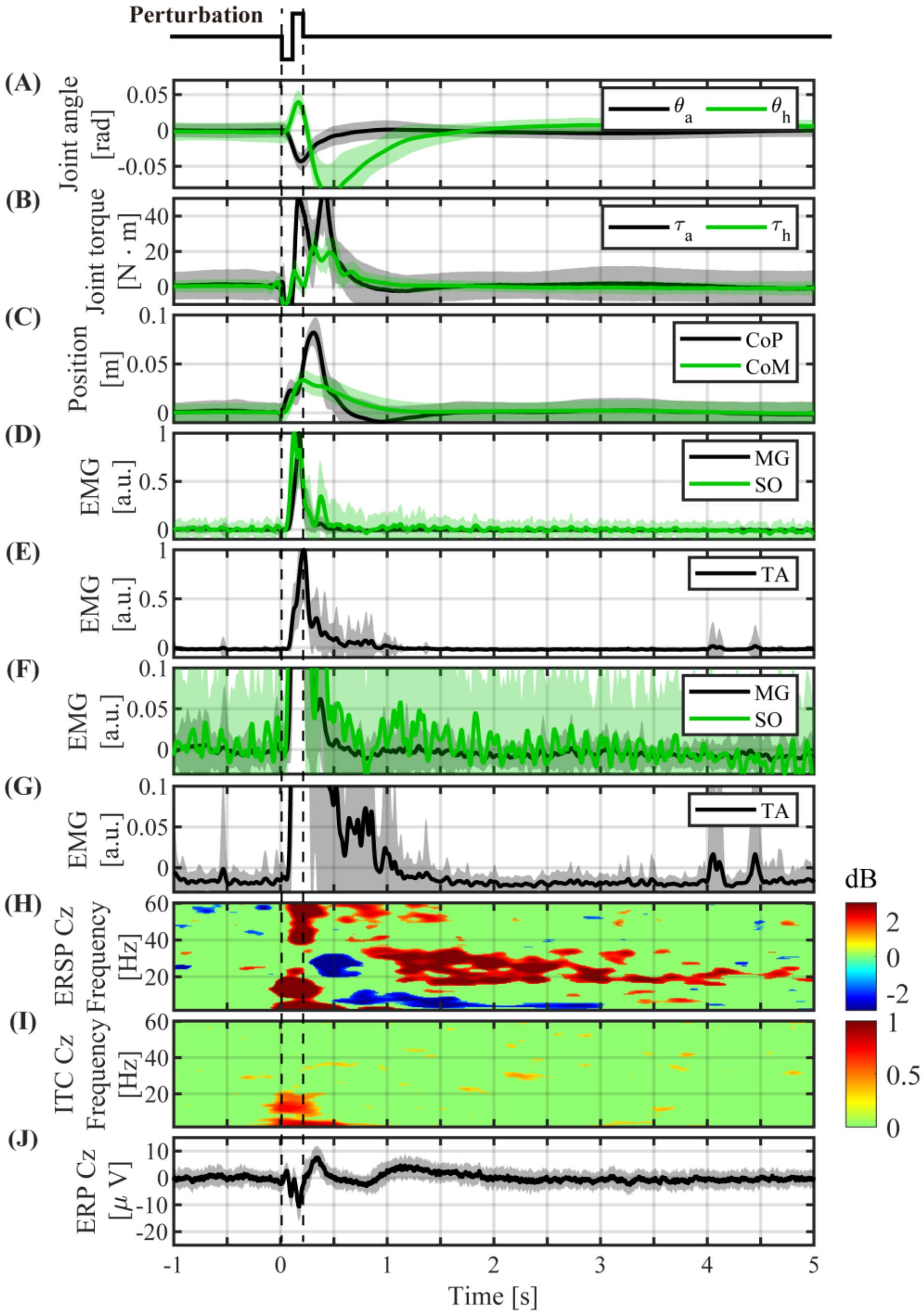

Figure S5. Event-locked average profiles of Subject 3 triggered by the perturbation-onset. (A) ankle and hip joint angles, (B) joint torques, (C) CoP and CoM positions, (D) normalized EMGs of Medial-Gastrocnemius and Soleus, (E) normalized EMG of Tibialis Anterior, (F) magnification of (D), (G) magnification of (E), (H) ERSP of Cz electrode, (I), ITC of Cz electrode, (J) ERP of Cz electrode. The light color shaded area in each of (A)-(G) and (J) is the standard deviation, representing the distribution across all perturbed trials of Subject 3. Nonsignificant differences from baseline (bootstrap statistics, $p > 0.05$) were set to 0 dB (green) in (H) and 0 (green) in (I).

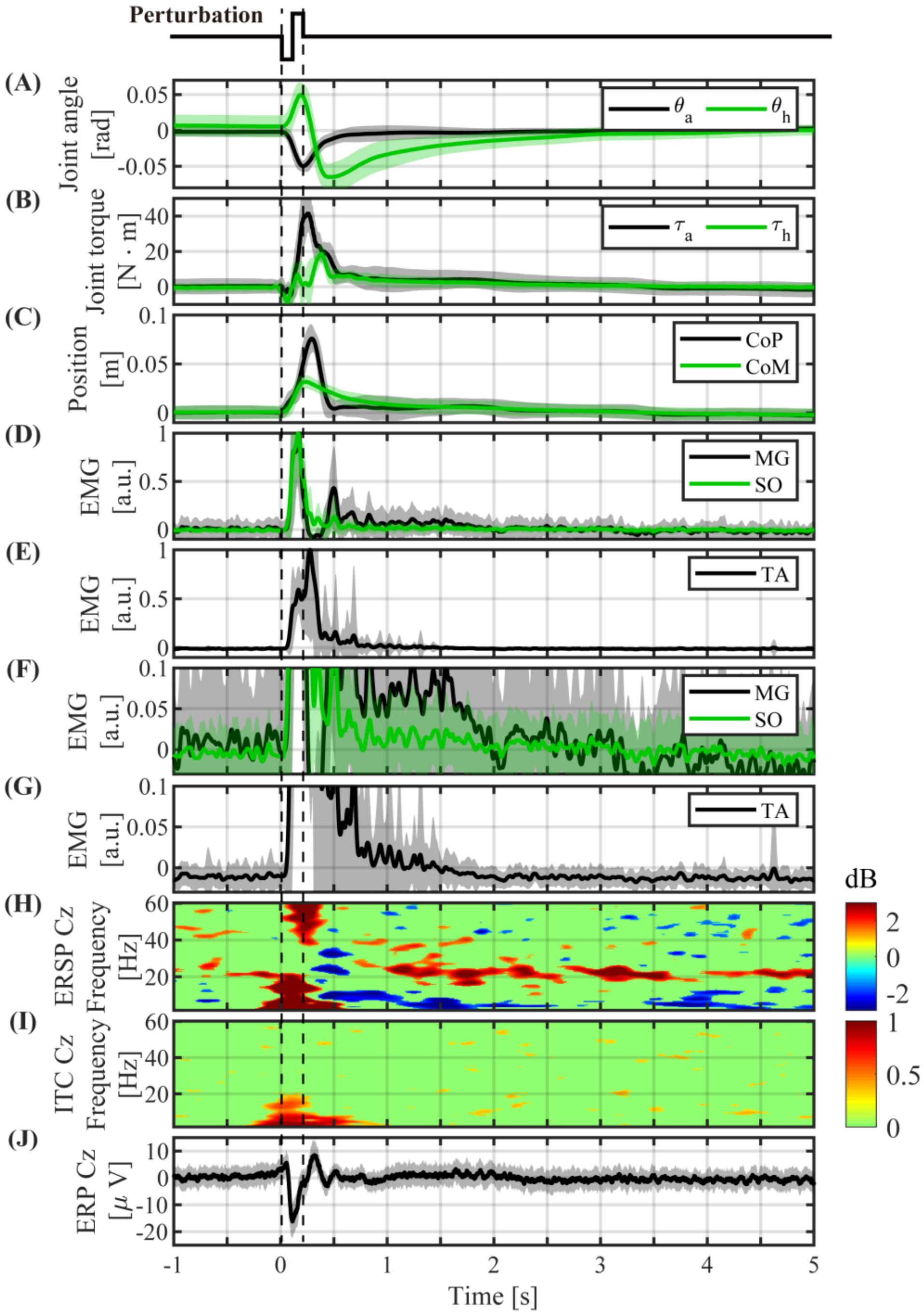

Figure S6. Event-locked average profiles of Subject 4 triggered by the perturbation-onset. (A) ankle and hip joint angles, (B) joint torques, (C) CoP and CoM positions, (D) normalized EMGs of Medial-Gastrocnemius and Soleus, (E) normalized EMG of Tibialis Anterior, (F) magnification of (D), (G) magnification of (E), (H) ERSP of Cz electrode, (I), ITC of Cz electrode, (J) ERP of Cz electrode. The light color shaded area in each of (A)-(G) and (J) is the standard deviation, representing the distribution across all perturbed trials of Subject 4. Nonsignificant differences from baseline (bootstrap statistics, $p > 0.05$) were set to 0 dB (green) in (H) and 0 (green) in (I).

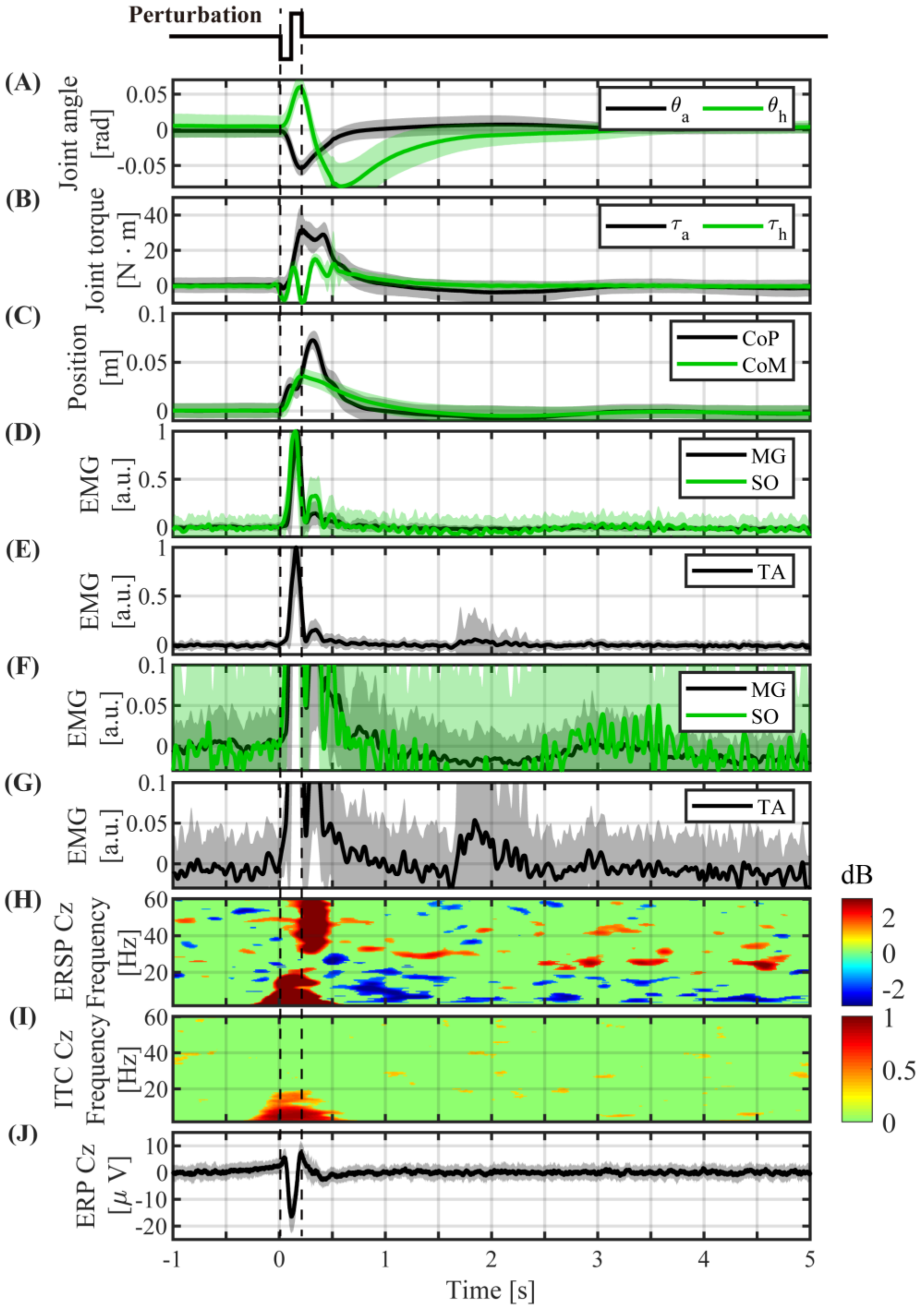

Figure S7. Event-locked average profiles of Subject 5 triggered by the perturbation-onset. (A) ankle and hip joint angles, (B) joint torques, (C) CoP and CoM positions, (D) normalized EMGs of Medial-Gastrocnemius and Soleus, (E) normalized EMG of Tibialis Anterior, (F) magnification of (D), (G) magnification of (E), (H) ERSP of Cz electrode, (I), ITC of Cz electrode, (J) ERP of Cz electrode. The light color shaded area in each of (A)-(G) and (J) is the standard deviation, representing the distribution across all perturbed trials of Subject 5. Nonsignificant differences from baseline (bootstrap statistics, $p > 0.05$) were set to 0 dB (green) in (H) and 0 (green) in (I).

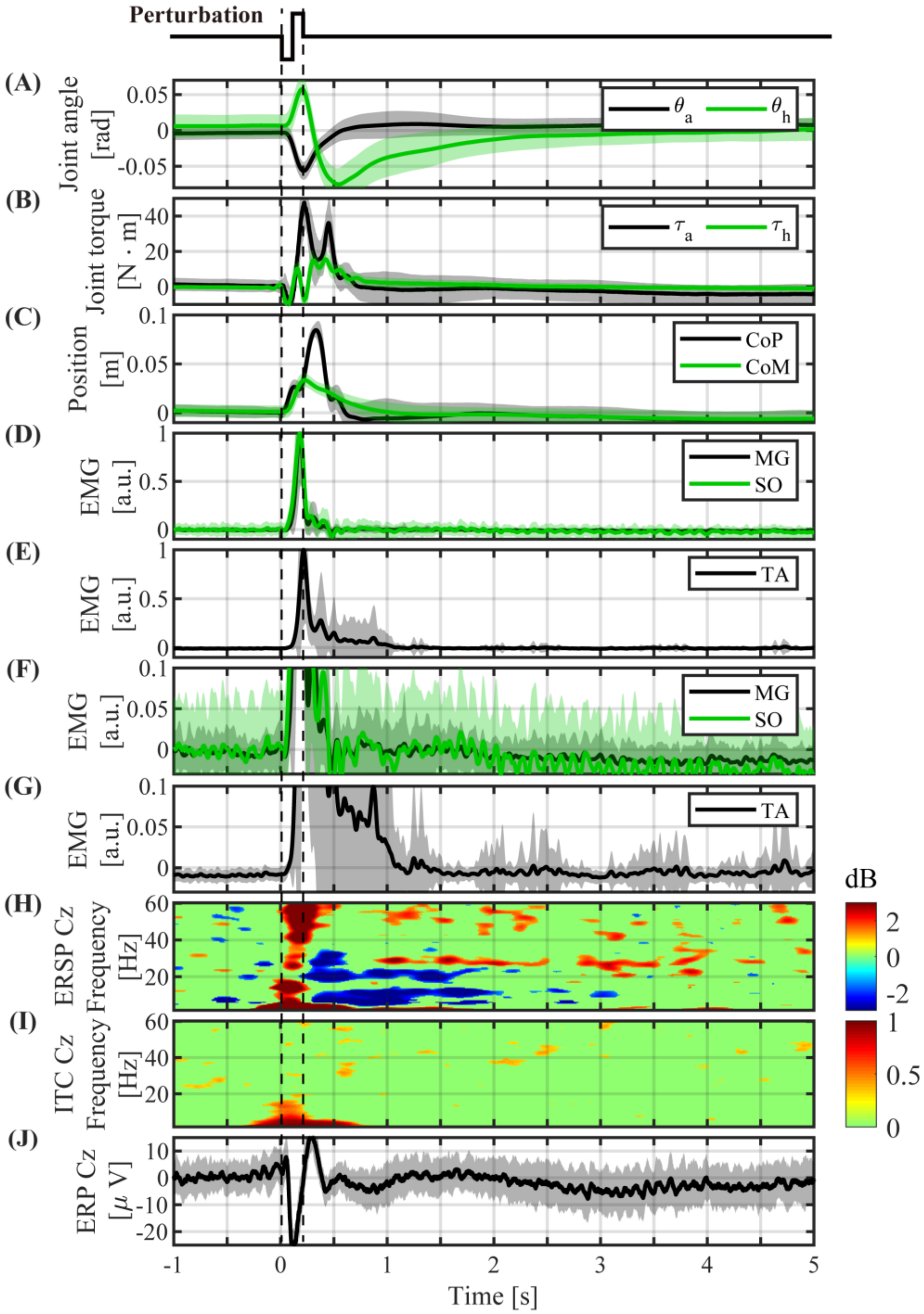

Figure S8. Event-locked average profiles of Subject 6 triggered by the perturbation-onset. (A) ankle and hip joint angles, (B) joint torques, (C) CoP and CoM positions, (D) normalized EMGs of Medial-Gastrocnemius and Soleus, (E) normalized EMG of Tibialis Anterior, (F) magnification of (D), (G) magnification of (E), (H) ERSP of Cz electrode, (I), ITC of Cz electrode, (J) ERP of Cz electrode. The light color shaded area in each of (A)-(G) and (J) is the standard deviation, representing the distribution across all perturbed trials of Subject 6. Nonsignificant differences from baseline (bootstrap statistics, $p > 0.05$) were set to 0 dB (green) in (H) and 0 (green) in (I).

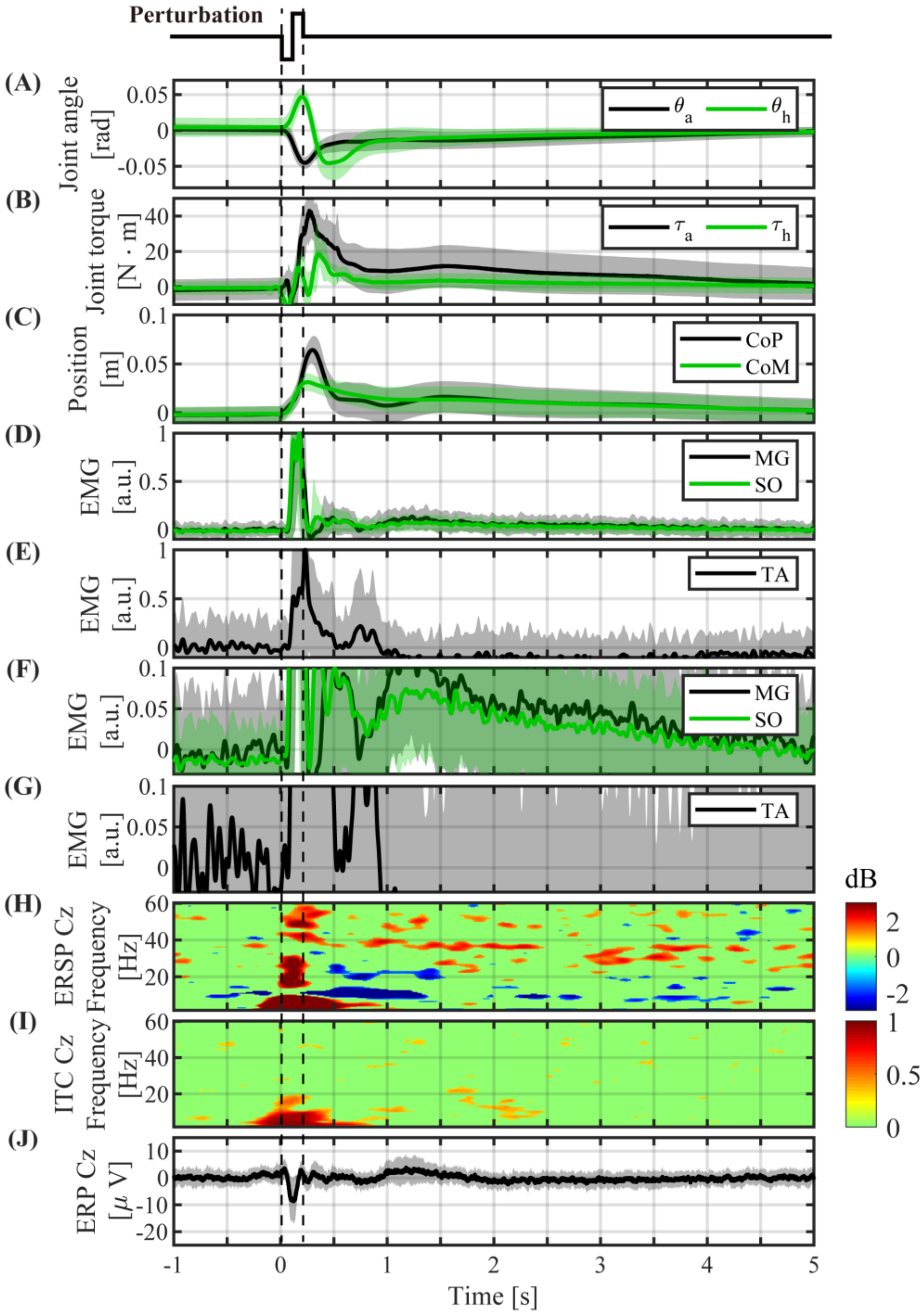

Figure S9. Event-locked average profiles of Subject 7 triggered by the perturbation-onset. (A) ankle and hip joint angles, (B) joint torques, (C) CoP and CoM positions, (D) normalized EMGs of Medial-Gastrocnemius and Soleus, (E) normalized EMG of Tibialis Anterior, (F) magnification of (D), (G) magnification of (E), (H) ERSP of Cz electrode, (I), ITC of Cz electrode, (J) ERP of Cz electrode. The light color shaded area in each of (A)-(G) and (J) is the standard deviation, representing the distribution across all perturbed trials of Subject 7. Nonsignificant differences from baseline (bootstrap statistics, $p > 0.05$) were set to 0 dB (green) in (H) and 0 (green) in (I).

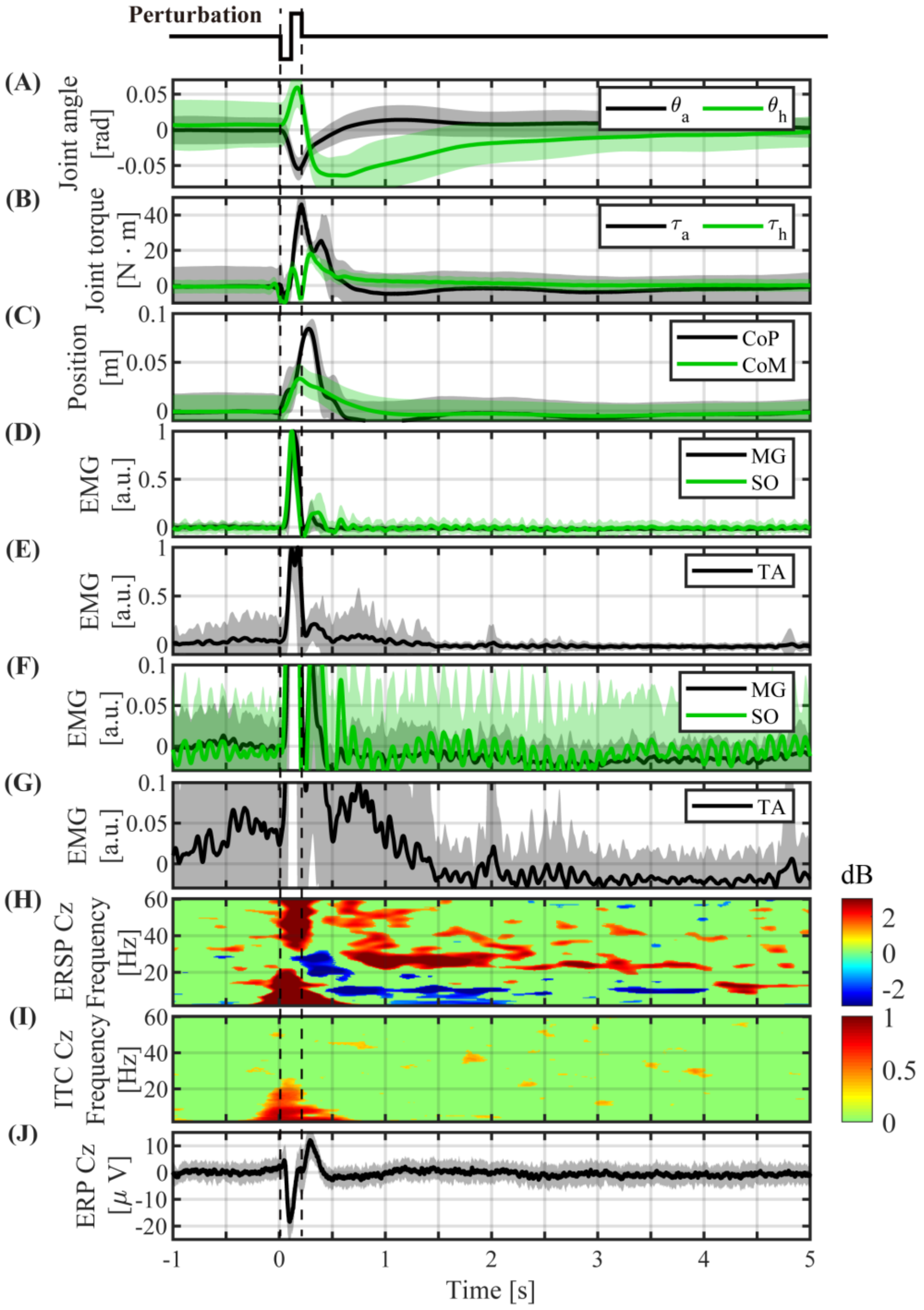

Figure S10. Event-locked average profiles of Subject 8 triggered by the perturbation-onset. (A) ankle and hip joint angles, (B) joint torques, (C) CoP and CoM positions, (D) normalized EMGs of Medial-Gastrocnemius and Soleus, (E) normalized EMG of Tibialis Anterior, (F) magnification of (D), (G) magnification of (E), (H) ERSP of Cz electrode, (I), ITC of Cz electrode, (J) ERP of Cz electrode. The light color shaded area in each of (A)-(G) and (J) is the standard deviation, representing the distribution across all perturbed trials of Subject 8. Nonsignificant differences from baseline (bootstrap statistics, $p > 0.05$) were set to 0 dB (green) in (H) and 0 (green) in (I).

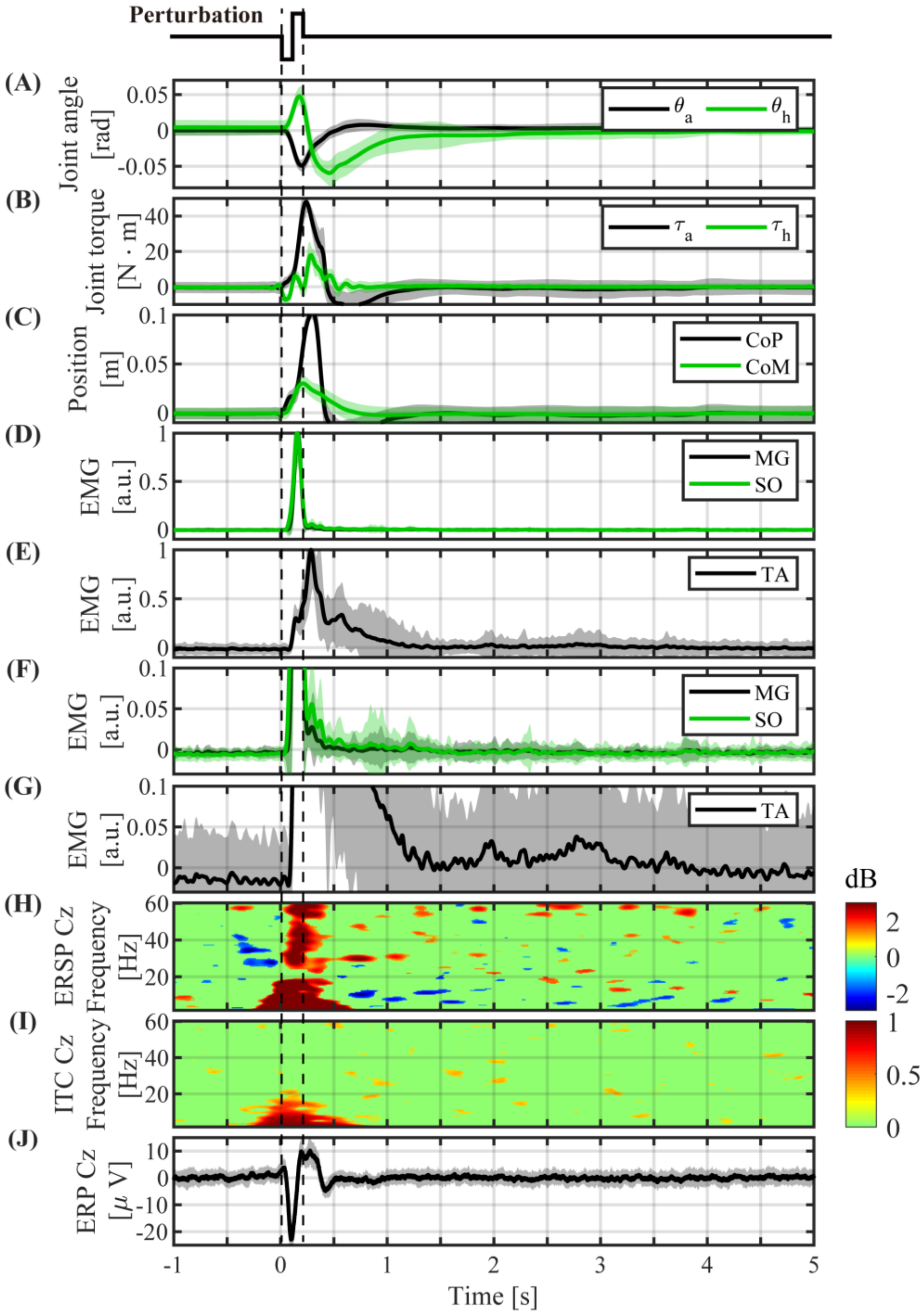

Figure S11. Event-locked average profiles of Subject 9 triggered by the perturbation-onset. (A) ankle and hip joint angles, (B) joint torques, (C) CoP and CoM positions, (D) normalized EMGs of Medial-Gastrocnemius and Soleus, (E) normalized EMG of Tibialis Anterior, (F) magnification of (D), (G) magnification of (E), (H) ERSP of Cz electrode, (I), ITC of Cz electrode, (J) ERP of Cz electrode. The light color shaded area in each of (A)-(G) and (J) is the standard deviation, representing the distribution across all perturbed trials of Subject 9. Nonsignificant differences from baseline (bootstrap statistics, $p > 0.05$) were set to 0 dB (green) in (H) and 0 (green) in (I).

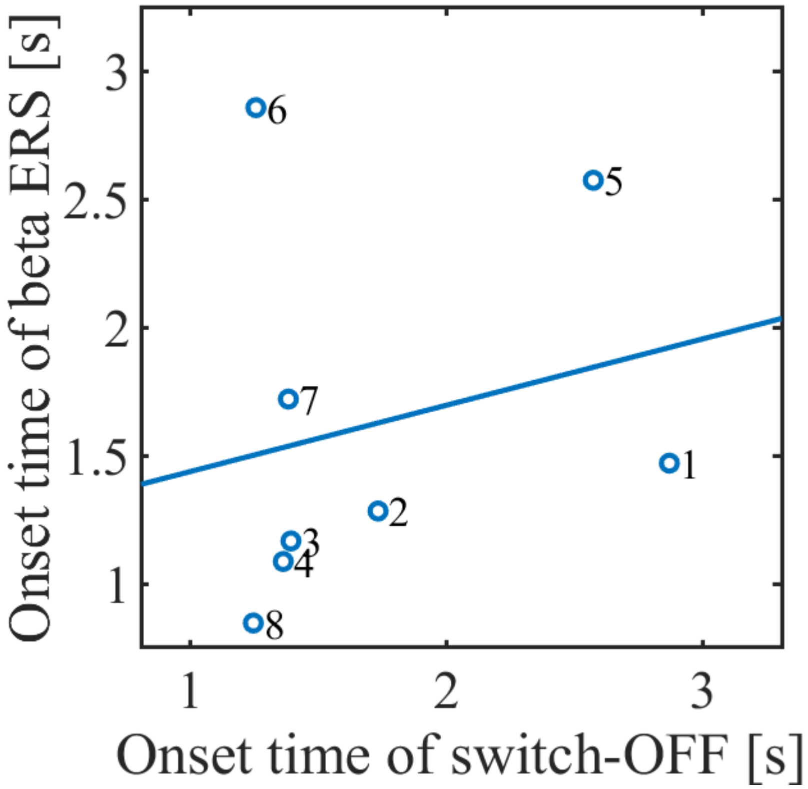

Figure S12. Correlation between the onset time of switch-OFF of the active controller and the onset time of the beta ERS. Each point represents those parameter values for each subject (1-8). Subject 9 was excluded from this analysis, because power of the beta ERS of Subject 9 was not large enough. The solid line represents the least square regression line. The correlation coefficient was 0.23.

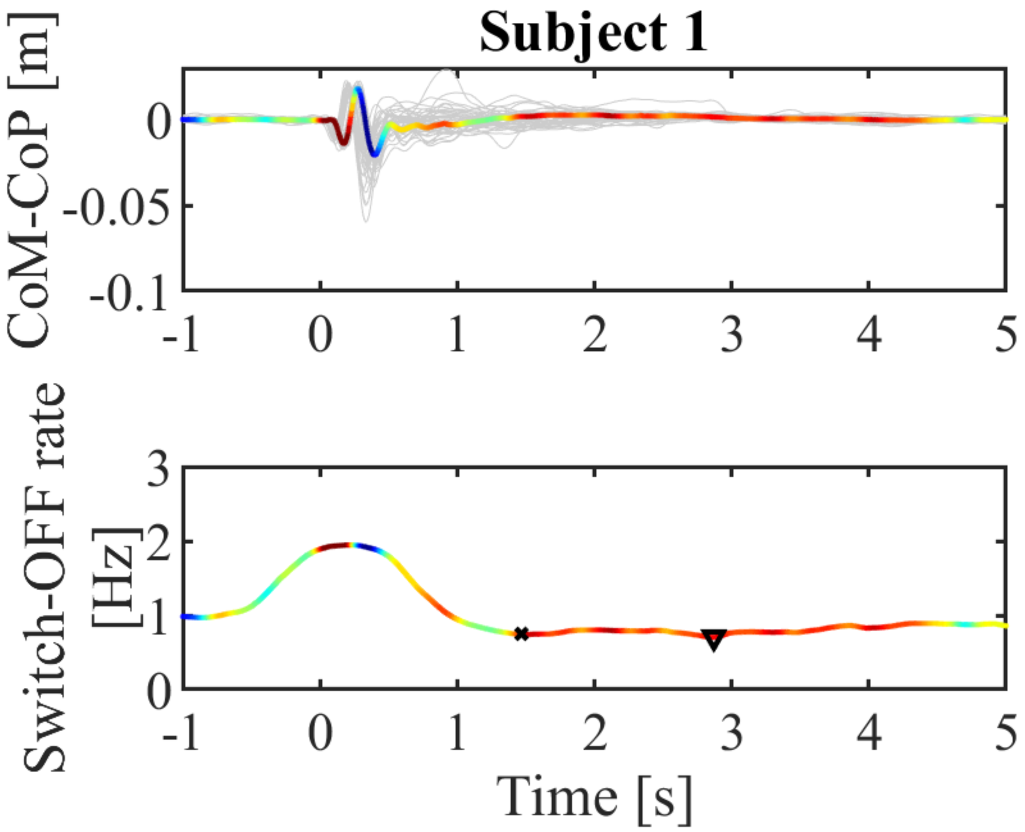

Figure S13. CoM-CoP time series and the switch-OFF rate time series for Subject 1. The upper panel plots the mean value of the CoM-CoP time series and the CoM-CoP time series (gray line) for each perturbation epoch. The line color for the averaged curve of CoM-CoP represents the power in the high-beta band of EEG. Red represents the ERS and blue represents the ERD. The lower panel is the switch-OFF rate (rate of the upward zero-crossing events for the CoM-CoP curve) as a function of time. As in the upper panel, the line colors represent high beta power of EEG. The lower triangular marker represents the time when the switch-OFF rate was minimum, which can be considered as the onset of switch-OFF of the active controller. The cross marker indicates the onset time of high-beta ERS.

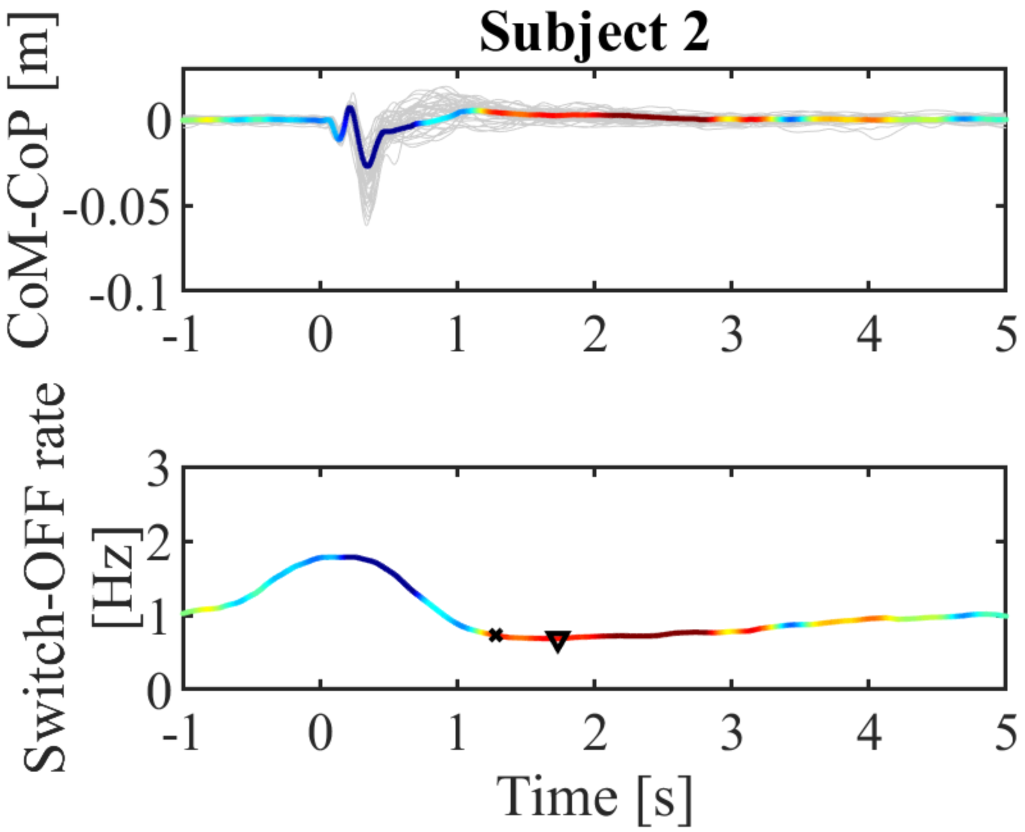

Figure S14. CoM-CoP time series and the switch-OFF rate time series for Subject 2. See the caption of Fig. S13.

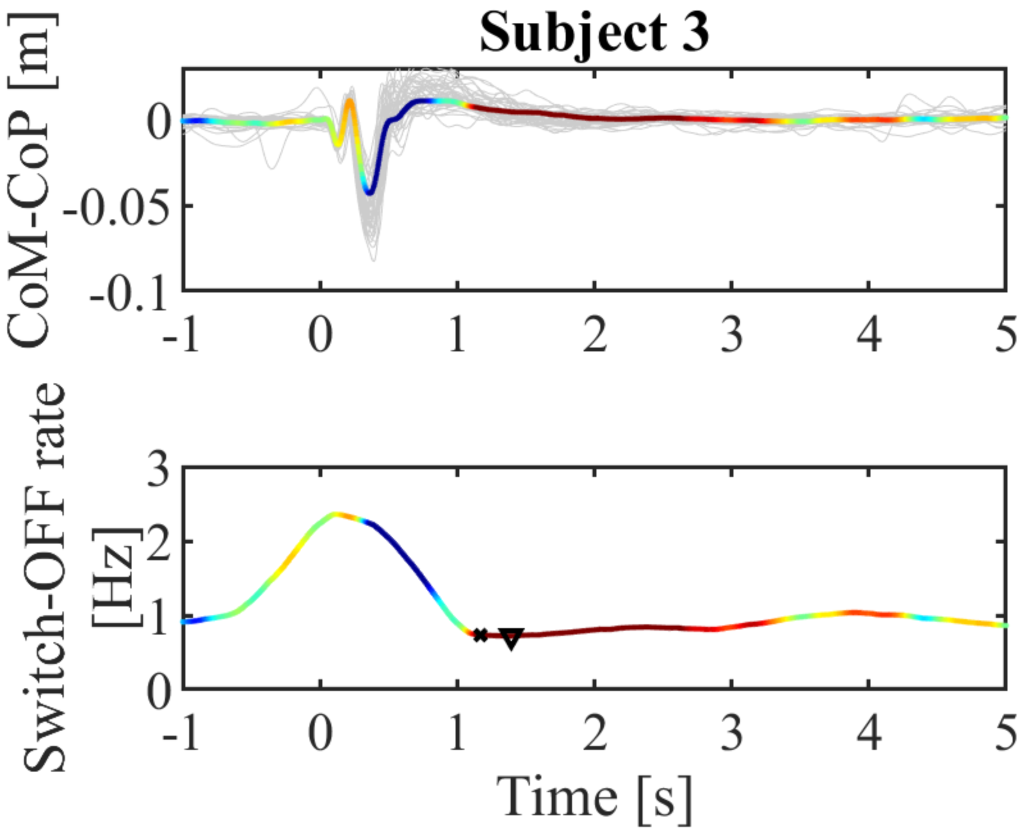

Figure S15. CoM-CoP time series and the switch-OFF rate time series for Subject 3. See the caption of Fig. S13.

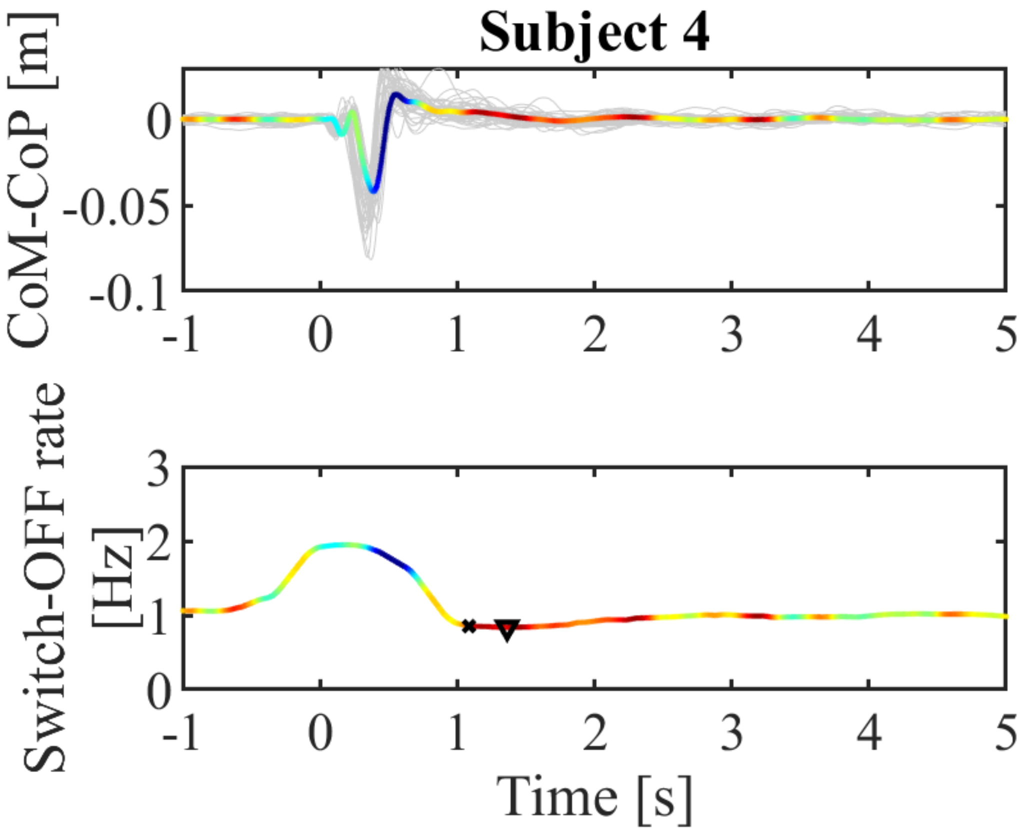

Figure S16. CoM-CoP time series and the switch-OFF rate time series for Subject 4. See the caption of Fig. S13.

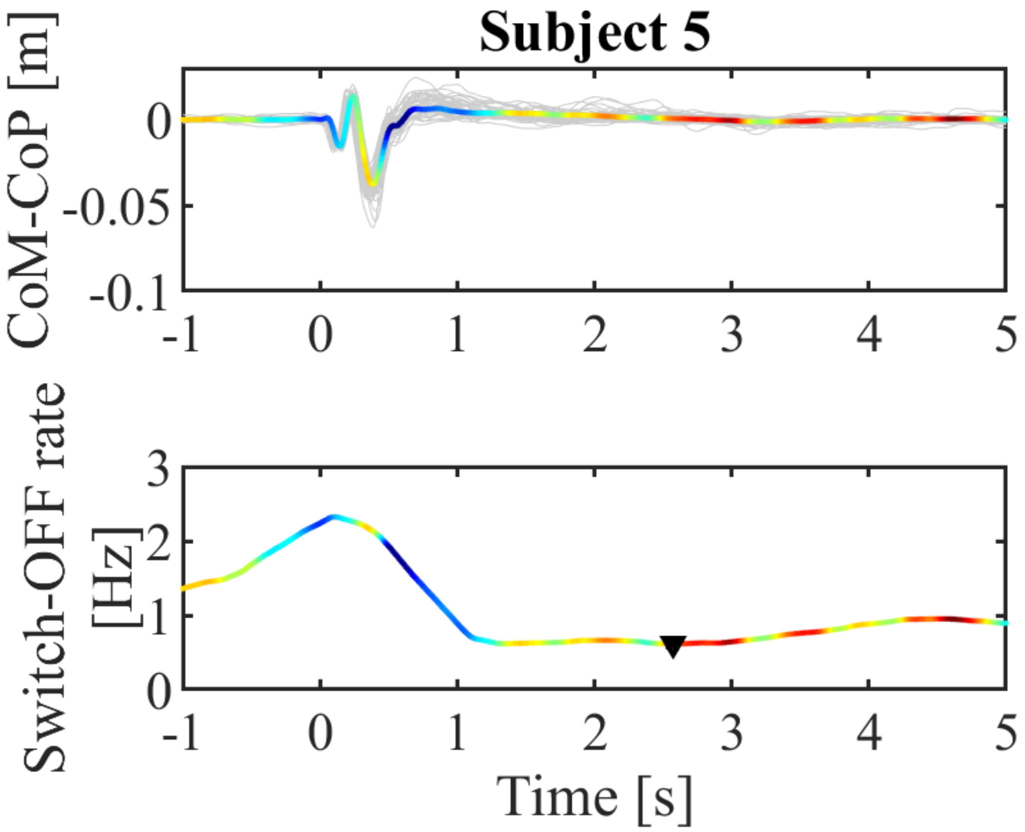

Figure S17. CoM-CoP time series and the switch-OFF rate time series for Subject 3. See the caption of Fig. S13.

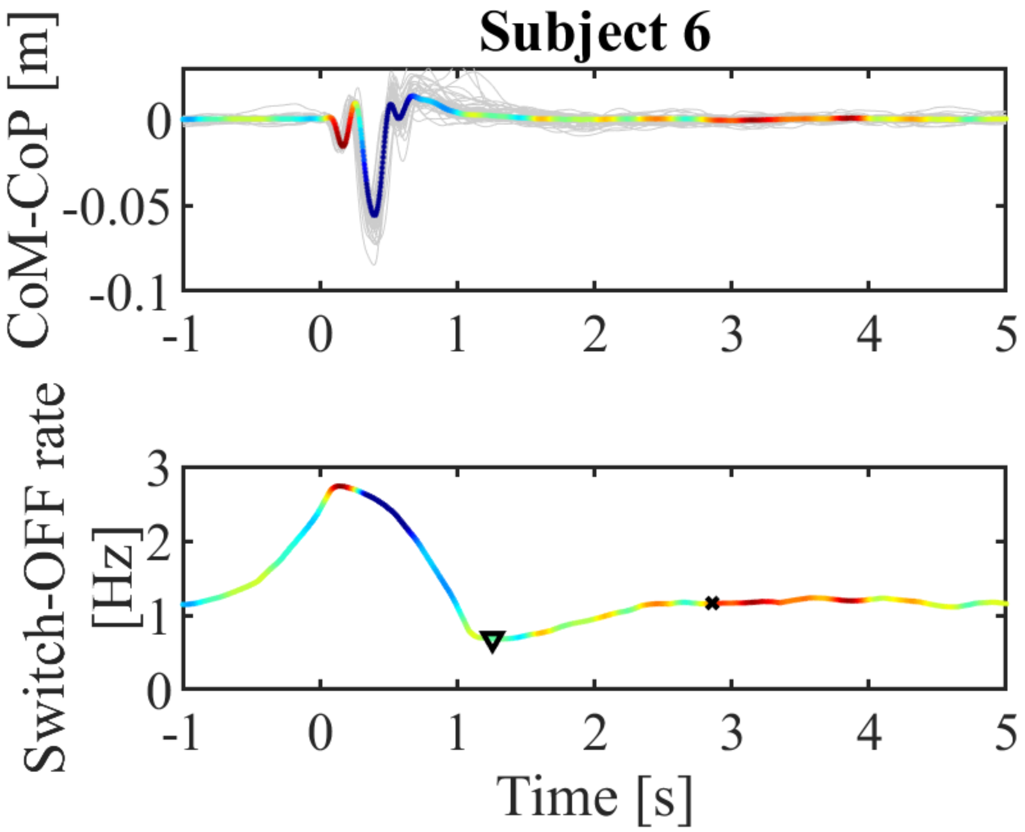

Figure S18. CoM-CoP time series and the switch-OFF rate time series for Subject 6. See the caption of Fig. S13.

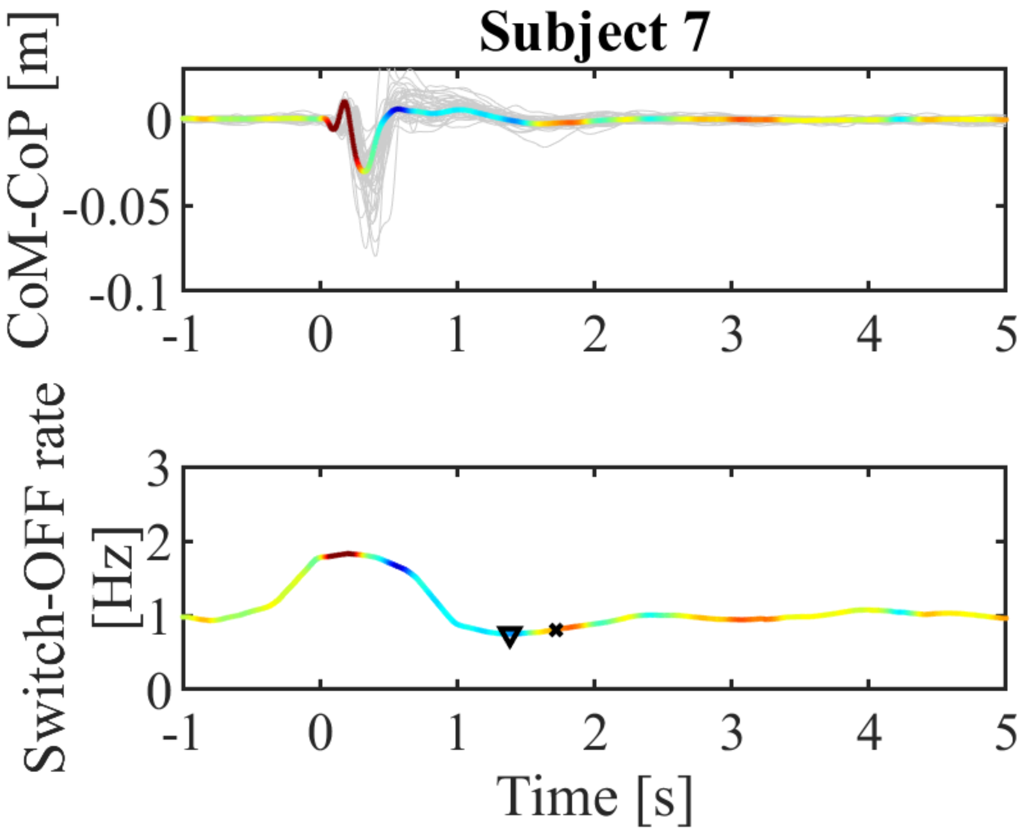

Figure S19. CoM-CoP time series and the switch-OFF rate time series for Subject 7. See the caption of Fig. S13.

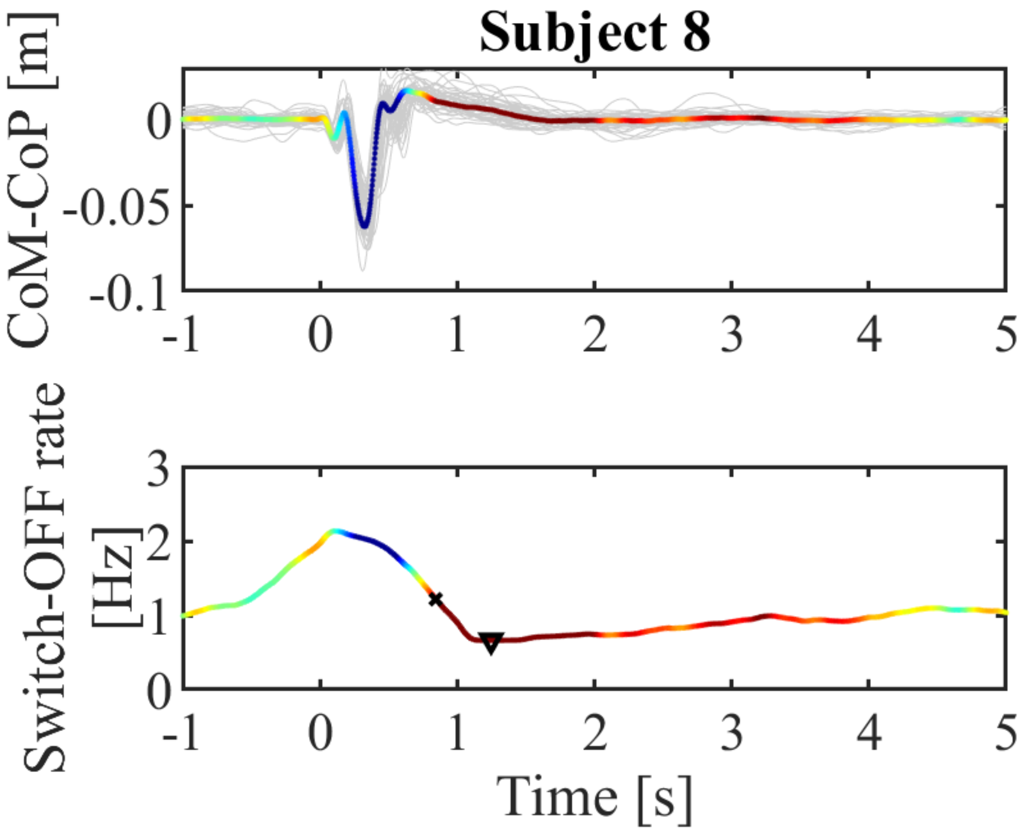

Figure S20. CoM-CoP time series and the switch-OFF rate time series for Subject 8. See the caption of Fig. S13.

References

Bigdely-Shamlo, N., Mullen, T., Kothe, C., Su, K.-M., and Robbins, K. A. (2015). The PREP pipeline: standardized preprocessing for large-scale EEG analysis. *Front. Neuroinformatics* 9. doi:10.3389/fninf.2015.00016.

Bruijn, S. M., Van Dieën, J. H., and Daffertshofer, A. (2015). Beta activity in the premotor cortex is increased during stabilized as compared to normal walking. *Front. Hum. Neurosci.* 9. doi:10.3389/fnhum.2015.00593.

Delorme, A., and Makeig, S. (2004). EEGLAB: an open source toolbox for analysis of single-trial EEG dynamics including independent component analysis. *J. Neurosci. Methods* 134, 9–21. doi:10.1016/j.jneumeth.2003.10.009.

Loo, S. K., Miyakoshi, M., Tung, K., Lloyd, E., Salgari, G., Dillon, A., et al. (2019). Neural activation and connectivity during cued eye blinks in Chronic Tic Disorders. *NeuroImage Clin.* 24, 101956. doi:10.1016/j.nicl.2019.101956.

Makeig, S. (1993). Auditory event-related dynamics of the EEG spectrum and effects of exposure to tones. *Electroencephalogr. Clin. Neurophysiol.* 86, 283–293. doi:10.1016/0013-4694(93)90110-H.

Morasso, P., Cherif, A., and Zenzeri, J. (2019). Quiet standing: The Single Inverted Pendulum model is not so bad after all. *PLoS ONE* 14, e0213870. doi:10.1371/journal.pone.0213870.

Mullen, T. R., Kothe, C. A. E., Chi, Y. M., Ojeda, A., Kerth, T., Makeig, S., et al. (2015). Real-time neuroimaging and cognitive monitoring using wearable dry EEG. *IEEE Trans. Biomed. Eng.* 62, 2553–2567. doi:10.1109/TBME.2015.2481482.

Suzuki, Y., Nomura, T., Casadio, M., and Morasso, P. (2012). Intermittent control with ankle, hip, and mixed strategies during quiet standing: A theoretical proposal based on a double inverted pendulum model. *J. Theor. Biol.* 310, 55–79. doi:10.1016/j.jtbi.2012.06.019.

Winkler, I., Debener, S., Müller, K., and Tangermann, M. (2015). On the influence of high-pass filtering on ICA-based artifact reduction in EEG-ERP. in *2015 37th Annual International Conference of the IEEE Engineering in Medicine and Biology Society (EMBC)*, 4101–4105. doi:10.1109/EMBC.2015.7319296.
